# Health and Development of Children Born Moderate and Late Preterm and Early Term at Age 10 in French Birth Cohorts ELFE and EPIPAGE 2

**DOI:** 10.1111/ppe.70069

**Published:** 2025-09-29

**Authors:** Laura Pavicic, Laetitia Marchand‐Martin, Ayoub Mitha, Marie‐Noelle Dufourg, Véronique Pierrat, Valérie Benhammou, Marie‐Aline Charles, Pierre‐Yves Ancel

**Affiliations:** ^1^ Université Paris Cité and Université Sorbonne Paris Nord, Inserm, INRAE, Center for Research in Epidemiology and Statistics (CRESS) Paris France; ^2^ Department of Neonatal Medicine, Bretonneau Hospital François Rabelais University Tours France; ^3^ Division of Clinical Epidemiology, Department of Medicine Solna Karolinska Institutet Stockholm Sweden; ^4^ UMS Elfe Team, Ined, Inserm, EFS Aubervilliers France; ^5^ Neonatal Intensive Care Unit Centre Hospitalier Intercommunal Créteil France

**Keywords:** birth cohort, health outcomes, morbidity, neurodevelopment, outcome‐wide, preterm birth

## Abstract

**Background:**

Lower gestational age (GA) is linked to higher mortality and morbidity. Long‐term health and developmental difficulties of individuals born moderate (MPT, 32–33 GA) and late (LPT, 34–36 GA) preterm, and early term (ET, 37–38 GA) are less explored than those of their very preterm peers.

**Objectives:**

To test how being born MPT, LPT, or ET affects health and development at age 10, compared to full‐term (FT, 39–40 GA) births.

**Methods:**

Data from two ongoing French nationwide birth cohorts, initiated in 2011, were collected at 10 years via telephone interview (*n* = 8372) and home visit (*n* = 6418). Weighting procedures accounted for study design, non‐inclusion, and participation. Outcome‐wide regressions (modified Poisson, linear), adjusted for socioeconomic situation and pregnancy complications, were used to calculate adjusted relative risks (aRR) and beta‐coefficients (*β*).

**Results:**

No increased risk of asthma/atopy was observed for our MPT, LPT, and ET populations, except for allergic rhinitis in MPT. Strabismus was more prevalent among MPT, LPT, and ET (2.3%–3.0%) than FT (1.3%), corresponding to aRR of 1.99 (95% CI 0.91, 4.39), 1.67 (95% CI 0.85, 3.28), and 2.18 (95% CI 1.37, 3.47), respectively. MPT and LPT had increased risk of balance problems, with aRR of 1.63 (95% CI 0.81, 3.32) and 1.80 (95% CI 1.14, 2.82), respectively. MPT scored on average lower on the WISC‐V full‐scale IQ Matrix *β* −0.6 (95% CI −1.17, −0.11) and performance IQ Puzzle *β* −0.7 (95% CI −1.23, −0.26) subtests, compared to FT, and had an increased risk of dental malposition, aRR 1.42 (95% CI 1.15, 1.75).

**Conclusions:**

While most outcomes (respiratory, anthropometry, cardiometabolic) did not differ between MPT, LPT, ET, and their FT peers, others, including strabismus, were more prevalent among preterm and ET. Some outcomes were specific to MPT, including lower WISC‐V average scores and dental issues.

## 1 Background

Preterm birth entails biological immaturity for extrauterine life and is defined as birth before 37 weeks of gestation (GA) [1]. It comprises four subgroups: extremely (< 28 GA), very (28–31 GA), moderate (32–33 GA), and late (34–36 GA) preterm [2]. Term birth is subdivided into early (37–38 GA), full (39–40 GA), and late (41 GA) term [3]. In 2020, global preterm birth rates ranged from 4% to 16%, with 5% to 10% prevalence in Europe [4, 5]. Gestational age is inversely associated with lifetime morbidity [5, 6], placing individuals born moderate (MPT) and late (LPT) preterm in a lower risk category than those born very preterm. Nonetheless, the final stages of lung, brain, and other tissue and organ prenatal development occur in the final weeks of pregnancy [7, 8, 9]. Consequently, MPT, LPT, and early term (ET) children face higher neonatal morbidity than their full‐term (FT) peers [10, 11], along with an increased risk of long‐term difficulties like suboptimal cognitive and motor development and impaired mental health [12, 13, 14, 15].

While a greater risk of neurodevelopmental and mental health impairments in childhood and adulthood is well documented among MPT, LPT, and ET [12, 13, 15, 16, 17, 18, 19, 20], other health outcomes such as atopy and puberty onset are less studied and often reported for preterm children as a single group [21, 22]. Understanding nuanced risks between MPT, LPT, ET, and FT births is crucial, particularly because MPT and LPT represent 85% of all preterm births [5], and some are electively planned [23]. This implies that the associated health risks might have been mitigated with delayed delivery. Similarly, assessing the long‐term risks associated with MPT, LPT, and ET births is important, as their high proportions imply meaningful potential gains if their prevalence or their long‐term risks are reduced with effective prevention.

We aimed to assess various health and developmental outcomes of MPT, LPT, and ET born children at age 10, compared to their FT peers.

## 2 Methods

### 2.1 Study Design and Setting

This study used data from two French nationwide birth cohorts, ELFE [24] (*Étude longitudinale française depuis l'enfance*) and EPIPAGE 2 [25] (*Étude épidémiologique sur les petits âges gestationnels*), both initiated in 2011. ELFE included single or twin live‐born infants at ≥ 33 GA to mothers having ≥ 18 years, with no plans to leave mainland France within 3 years [26]. EPIPAGE 2 included all live births, stillbirths, or pregnancy terminations between 22 and 31 GA, along with a sample of preterm infants born at 32–34 GA [25]. No participants were included in both cohorts. Detailed study protocols are described elsewhere [25, 26].

ELFE recruited from 320 maternity units across mainland France on 25 selected days across four seasons [26], while EPIPAGE 2 enrolled participants born at 32–34 GA from all maternity units in 25 French regions over 5 weeks (May 2–June 5, 2011) [25]. Baseline data were collected at birth and 2 months for ELFE, and at birth and during neonatal unit admission for EPIPAGE 2. A uniform follow‐up at age 10.5^1^ years was predefined in the follow‐up protocol of both cohorts and consisted of two data collection components: a telephone interview and a home visit.

### 2.2 Ethics Statement

ELFE: Informed consent for participation was signed at birth by both parents or mother alone, with father informed of his right to deny consent. EPIPAGE 2: Data collection occurred after families had received information and agreed to participate in the study. At the 10‐year follow‐up for both cohorts, one parent signed an informed consent and attested that the second parent had been informed about the child's participation, while children were asked for an oral consent.

As required by French law and regulations, the National Committee on Information Processing in Health Research (*Comité Consultatif sur le Traitement de l'information en matière de Recherche dans le Domaine de la Santé*), the National Data Protection Authority (*Commission Nationale Informatique et Liberté*), and the Committee for Protection of Persons Engaged in Research (*Comité de Protection des Personnes*) approved both cohorts.

### 2.3 Variables and Data Sources/Measurements

#### 2.3.1 Exposure—Gestational Age

Gestational age, defined as the best obstetric estimate combining last menstrual period and ultrasonogram from medical files, was a continuous variable in weeks of gestation. We categorised it into 4 groups (FT, 39–40 GA; MPT, 32–33 GA; LPT, 34–36 GA; ET, 37–38 GA).

#### 2.3.2 Outcomes—Health and Development at 10 Years

Outcomes were collected at 10 years in two phases. First, parents completed a telephone interview covering their child's health and development, including: respiratory difficulties and atopy (allergic rhinitis, eczema, food allergies), signs of puberty (thelarche, pubarche, menarche), visual and dental disorders, behavioural difficulties (Strengths and Difficulties Questionnaire (SDQ) [27]—a 25‐item screening tool for ages 4–17), common physical complaints, and timing and adequacy of sleep. The history of asthma variable was constructed using the Mechanisms of the Development of Allergy (MeDALL) criteria [28]. The history of allergic rhinitis and eczema was based on parent‐reported diagnosis (ever). The food allergy variable was constructed from multiple questions assessing the elimination of foods from a child's diet since age 1, following a paediatrician's advice. All variables were categorised as either ‘yes’ (presence of the condition) or ‘no’ (absence), except for pubarche, which had three categories due to 16% of parents reporting uncertainty about whether their children had pubic hair. The SDQ variables were dichotomised to compare the scores categorised as ‘at risk’ with the two other score categories, ‘normal’ and ‘borderline’; details on scoring and categorisation are described elsewhere [27].

Second, during a home visit, interviewers collected the following measurements: weight, height, waist circumference, blood pressure (BP), heart rate (HR), tests assessing motor and cognitive development (described below). Waist‐to‐height ratio z‐scores were calculated using the population born FT as the reference, while the z‐scores for weight, height, and body mass index (BMI) for age and sex were calculated following the World Health Organisation's Child Growth Standards [29]. BMI was calculated and categorised (underweight, normal weight, overweight, obesity) using the International Obesity Task Force (IOTF) cut‐offs [30, 31]. Two BP and HR readings were taken with the automatic oscillometer ‘Omron 705IT’ on the child's left upper arm—the first after a 3–5 min rest and the second 2 min later. To account for the ‘white coat’ effect, the second reading was used to determine systolic and diastolic BP percentiles and classify BP level (normal, pre‐elevated, elevated) per American Academy of Paediatrics cut‐offs [32]. HR was averaged from two readings. Motor skills were assessed via four modules (dribbling, ball throwing, one‐leg exercises, and jumping), each comprising 4–5 exercises from the Test of Gross Motor Development 2 [33]. Exercises were rated as successful, unsuccessful, or not observed and assigned scores of 1, 0, or missing, respectively. We summed the number of successful exercises per module and converted them into binary variables with cut‐offs at ≤ 2/4 or ≤ 2/5 successful exercises vs. higher performance. Similarly, a global score combining all modules with a cut‐off at ≤ 8/17 was used to identify low performers. Physical fitness was assessed via the Eurofit Fitness Testing Battery [34] (sits‐ups in 30 s, jump length). Intellectual and cognitive skills were evaluated using the matrix and puzzle subtests from the Wechsler Intelligence Scale for Children V (WISC‐V) [35, 36], as well as children's knowledge of spoken words and receptive vocabulary from the Peabody Picture Vocabulary Test (PPVT) 5 [37, 38] adapted to French. The matrix subtest of the Fluid Reasoning Index assesses problem‐solving and perceptual organisation, while the puzzle subtest of the Visual Spatial Index evaluates non‐verbal reasoning, spatial processing, and attention to detail [36]. Standard subtest scores (mean = 10, SD = 3) [39] were used as continuous variables in the analyses.

#### 2.3.3 Covariates/Confounders

Following the instructions for outcome‐wide studies [40], we identified a common set of confounders to adjust for across all analyses. Based on a thorough literature review, we constructed a directed acyclic graph (DAG) using DAGitty [41] (Appendix S1) and adjusted for the following pre‐exposure variables: mother's age at birth, parental socioeconomic factors at pregnancy (maternal education and employment, household income, and the highest socio‐professional category between two parents), mother's country of birth, smoking during pregnancy, mother's medical history (diabetes mellitus, arterial hypertension, infertility treatment, pre‐pregnancy BMI), foetal growth restriction or small‐for‐gestational‐age diagnosed during pregnancy (reported and/or extracted from medical files), and child's sex. Maternal mental health during pregnancy was identified as a confounder [42, 43], but could not be harmonised across cohorts, so we replicated analyses in the ELFE subsample to assess whether our results or conclusions changed with additional adjustment. We also replicated analyses in the subsample of singletons (ELFE and EPIPAGE 2) to account for differences between twin and singleton births. Since our conclusions remained consistent, we present results from the full sample; subsample analyses results are in the Appendix S1.

### 2.4 Study Size/Participants

**FIGURE 1:**
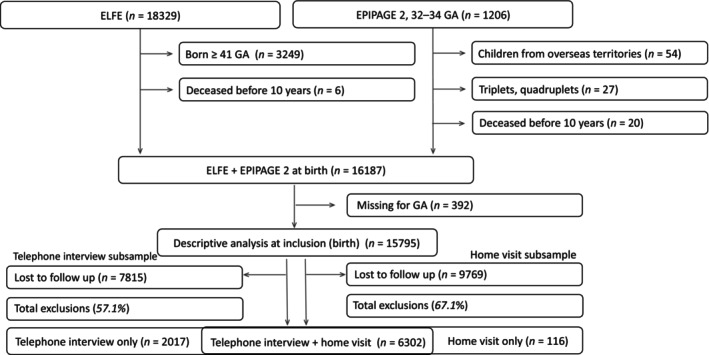
Study sample flowchart.

We included the ELFE participants born at 33–40 GA and those from EPIPAGE 2 born at 32–34 GA, excluding stillbirths, deaths before the age of 10, EPIPAGE 2 participants from French overseas territories (ELFE did not recruit there), and EPIPAGE 2 triplets and higher‐order pregnancies (not included in ELFE). After merging the cohorts, the total sample at inclusion was *N* = 16,187. We applied a weighting procedure, accounting for the cohort design, initial non‐inclusion, and 10‐year participation, separately for the sample at inclusion, telephone interview, and home visit. The final weights were calculated as a product of sampling, initial non‐inclusion, and 10‐year participation weights aimed at reconstituting the population of singleton and twin children born in mainland France in 2011 at 32–40 GA. The weighting for the two cohorts is detailed elsewhere [44, 45, 46]. Due to attrition, the subsample sizes at 10 years were *n* = 8372 for the telephone interview and *n* = 6418 for the home visit.^2^ Figure 1 illustrates our sample size flowchart before imputations.

### 2.5 Missing Data

Missing data at inclusion ranged from 0.3% to 22.0%. For the telephone interview, missing data ranged from 1.0% to 6.5%, and for the home visit from 0.1% to 7.3%. Percentages missing for the outcomes are listed in the Appendix S1. Given the differing sample sizes, we imputed the telephone interview and home visit subsamples separately, using a hybrid method that combined multiple imputations by chained equations with random forest (via the ‘*miceRanger*’ [47] package) to generate 50 datasets each. In line with Van Buuren's [48] advice for large datasets with high multicollinearity between variables, we limited our predictor variables to < 15 (Appendix S1). These were selected based on significant differences between exposure groups and minimal missing data, as determined via descriptive analyses.

### 2.6 Statistical Analysis

We used R (version R 4.2.1) via RStudio to conduct analyses. Weights were applied through the ‘*survey*’ [49] package and a weighted pseudo‐population used throughout analyses. We described the sample at birth using percentages and counts, % (*n*) for categorical variables, and means (± SD) for continuous variables. To test associations between GA and health and developmental outcomes at 10 years, we employed the outcome‐wide multivariable regression [40] using cluster‐robust variance estimate [50] to account for unmeasured dependence between twins from the same family. The modified Poisson regression was used for binary outcomes, linear for continuous, and multinomial logistic regression for outcomes having ≥ 3 categories before dichotomisation, or challenging to dichotomise (pubarche (ref no), SDQ score (ref normal), IOTF BMI (ref normal weight), anthropometric z‐scores (ref 0), BP level (ref normal)). Bonferroni corrections were not used to correct for multiple testing, as they impose severe penalties and could significantly increase the risk of type II error when the number of analysed outcomes is large [51]. Although the total study sample might be large enough to handle the corrections, the 4 groups of GA have very heterogeneous sample sizes and power to identify true positive results. Sensitivity analyses for unmeasured confounding [40] were conducted using the ‘*E‐Value*’ [52] package. Results are presented as RR, 95% CI, *β*, 95% CI, and OR, 95% CI. We report and interpret prevalence and associated risk across GA groups in the context of existing knowledge, rather than relying solely on significance levels, which are highly sample‐size dependent.

## 3 Results

### 3.1 Descriptive Data

We observed a lower education level for mothers of LPT and ET infants, compared to FT (Table 1). Socioeconomic variables were more heterogeneous for mothers of MPT infants, with education level like mothers of FT, but a higher frequency of unemployment and stay‐at‐home parents. We also observed important differences across GA groups for diabetes mellitus, arterial hypertension, and infertility treatment, which were all more prevalent among mothers of MPT and LPT infants, compared to those of FT infants. MPT, LPT, and ET infants were more often born via elective caesarean, from twin pregnancies, and small for gestational age, compared to those born FT. Descriptive analyses before weighting are presented in Table S1.

**TABLE 1:** Weighted descriptive analysis of socioeconomic factors and maternal comorbidities, collected at birth (inclusion) from ELFE and EPIPAGE 2 cohorts combined; *n* = 16,187.

Characteristic	Missing	MPT, 32–33 GA, *n* = 656^*a*^	LPT, 34–36 GA, *n* = 1438^*a*^	ET, 37–38 GA, *n* = 3728^*a*^	FT, 39–40 GA, *n* = 9973^*a*^
**Mother's age at birth**	0.5% (106)				
≤ 24		10.4% (75)	18.1% (223)	15.2% (485)	16.4% (1352)
25–34		68.3% (435)	58.9% (886)	63.0% (2438)	65.0% (6694)
≥ 35		21.3% (146)	23.0% (329)	21.8% (800)	18.7% (1916)
**Mother's country of birth**	1.0% (142)				
France		80.5% (534)	84.1% (1226)	80.0% (3188)	82.0% (8665)
Other European countries		2.8% (18)	2.4% (39)	1.9% (81)	2.5% (225)
North African countries		5.5% (38)	6.0% (72)	8.3% (195)	7.7% (478)
Other African countries		7.4% (34)	4.3% (58)	7.0% (170)	5.0% (346)
Other countries		3.8% (27)	3.3% (39)	2.8% (82)	2.8% (230)
**Mother's education level**	0.7% (210)				
≤ Primary education		10.6% (52)	13.6% (140)	13.9% (330)	11.8% (717)
High school		37.9% (235)	43.1% (571)	42.7% (1487)	40.1% (3707)
≤ 2 years of University		17.9% (128)	16.7% (275)	15.3% (677)	16.2% (1845)
> 2 years of University		33.7% (203)	26.5% (402)	28.2% (1229)	31.9% (3691)
**Mother's employment**	5.7% (580)				
Professional activity		63.4% (434)	66.1% (1021)	63.9% (2735)	67.9% (7576)
Unemployed		9.9% (58)	7.9% (101)	7.6% (238)	6.6% (549)
Stay at home parent		20.0% (101)	17.4% (169)	19.0% (385)	16.4% (908)
Student		1.8% (15)	3.3% (33)	3.6% (94)	4.1% (263)
Other		4.9% (28)	5.4% (65)	5.9% (177)	5.0% (413)
**Household CSP** ^b^	4.4% (360)				
Executive		22.2% (147)	21.7% (322)	20.7% (958)	24.1% (2846)
Middleman professions		16.3% (109)	17.1% (280)	18.3% (781)	19.4% (2191)
Administrative, public service, student		13.2% (82)	12.1% (144)	11.3% (335)	11.5% (913)
Service job, commerce		35.6% (225)	40.2% (533)	40.7% (1435)	37.5% (3546)
Manual worker		11.4% (59)	6.4% (92)	8.5% (157)	6.5% (314)
Without profession		1.1% (7)	2.5% (20)	0.7% (10)	1.0% (34)
**Household income** ^c^	22% (2564)				
< 1500 €		15.9% (67)	14.0% (136)	11.9% (260)	11.3% (739)
[1500€—4000 €]		65.8% (319)	68.6% (780)	65.4% (2092)	64.9% (5544)
> 4000 €		18.3% (96)	17.4% (212)	22.7% (818)	23.8% (2318)
**Mother's pre‐pregnancy BMI**	2.9% (407)				
< 18.5		10.4% (65)	10.5% (143)	8.3% (304)	8.2% (802)
18.5–24.9		57.3% (384)	60.4% (900)	61.2% (2322)	64.5% (6491)
25.0–29.9		19.3% (115)	15.0% (208)	18.5% (642)	17.2% (1624)
≥ 30.0		13.0% (75)	14.1% (162)	12.0% (399)	10.1% (901)
**Gestational DM** ^d^	5.3% (989)	13.4% (76)	10.3% (147)	11.3% (403)	6.7% (650)
**History of DM**	3.1% (591)	2.7% (9)	2.3% (29)	2.0% (67)	0.7% (71)
**Gestational HTA** ^e^	2.8% (573)	23.3% (140)	11.0% (186)	5.5% (183)	2.9% (255)
**History of HTA**	2.4% (464)	6.9% (37)	5.2% (82)	3.0% (103)	2.2% (202)
**Infertility treatment**	2.4% (392)	18.2% (132)	12.7% (220)	7.4% (346)	6.2% (709)
**Tobacco smoking** ^f^	2.1% (338)	18.1% (119)	26.7% (343)	24.5% (822)	20.8% (1927)
**Foetal growth restriction** ^g^	4.5% (838)	21.1% (128)	12.4% (178)	5.5% (194)	2.5% (225)
**Labour type**	2.6% (514)				
Vaginal delivery		32.9% (224)	55.9% (736)	65.7% (2355)	72.3% (7144)
Vaginal instrumental delivery		5.7% (40)	6.7% (107)	8.7% (367)	12.4% (1262)
Elective caesarean		45.2% (274)	24.3% (387)	17.0% (639)	7.3% (675)
Non‐elective caesarean		16.2% (115)	13.0% (190)	8.5% (304)	8.0% (761)
**Multiple births** (Twins)	0.3% (59)	37.5% (252)	24.5% (382)	7.9% (296)	0.3% (32)
**Resuscitation at birth** (Yes)	1.7% (280)	39.4% (260)	12.7% (228)	3.5% (118)	2.3% (212)
**Child's sex** (Female)	0.6% (99)	49.4% (303)	46.9% (679)	47.4% (1757)	49.5% (4922)
**Birth weight *z*‐score** ^h^	3.6% (585)				
< −2 SD		19.4% (130)	10.8% (154)	3.6% (133)	1.8% (189)
[−2 SD, −1 SD]		24.8% (150)	19.2% (273)	14.6% (536)	12.9% (1260)
[1SD, 2 SD]		5.6% (36)	9.6% (129)	12.9% (499)	14.0% (1327)
≥ 2 SD		2.1% (14)	6.4% (55)	5.1% (199)	3.9% (372)
**Exclusive breastfeeding**	1.7% (271)	36.7% (238)	35.9% (556)	54.0% (2054)	60.1% (6196)
**Breast feeding duration** ^i^	21% (2464)	1.71 (3.11)	2.22 (4.87)	2.99 (5.12)	3.28 (5.42)

^*a*^ % (*n* (unweighted)); mean (SD).

^b^ CP: from French catégorie socioprofessionnelle, meaning socio‐professional domain.

^c^ Household income per month.

^d^ DM: Diabetes mellitus.

^e^ HTA: arterial hypertension.

^f^ Tobacco smoking during the second half of pregnancy.

^g^ Intrauterine growth restriction suspected during pregnancy.

^*h*^ Gardosi birth weight *z*‐scores.

^i^ Breast feeding duration in months, variable collected at multiple follow‐ups between 0 and 2 years and harmonised between cohorts.

### 3.2 Main Results

#### 3.2.1 Vision

Participants born MPT, LPT, and ET had a higher risk of strabismus at age 10 compared to FT peers, with respective aRR of 1.99 (95% CI 0.91, 4.39), 1.67 (95% CI 0.85, 3.28), and 2.18 (95% CI 1.37, 3.47) (Table 2). Similarly, children born LPT had 9%–38% greater risk of wearing glasses and around 40% increased risk of being followed by an ophthalmologist for astigmatism and/or hyperopia. Neither MPT nor ET showed increased risk for either of these disorders. Conversely, MPT had around 30% higher risk of myopia compared to FT.

**TABLE 2:** Descriptive analysis (prevalence) and outcome‐wide regression results of reported outcomes from the weighted telephone interview subsample, *n* = 8372.

Outcomes	Gestational age															
	39–40 GA, *n* = 5466	32–33 GA, *n* = 328					34–36 GA, *n* = 680					37–38 GA, *n* = 1898				
			Unadjusted		Adjusted			Unadjusted		Adjusted			Unadjusted		Adjusted	
	%	%	RR	95% CI	RR	95% CI	%	RR	95% CI	RR	95% CI	%	RR	95% CI	RR	95% CI
**Respiratory and allergies**	(1.00)															
Asthma	6.2%	8.4%	1.35	0.82, 2.22	1.22	0.71, 2.10	8.6%	1.39	0.91, 2.11	1.23	0.84, 1.79	7.7%	1.24	0.96, 1.58	1.20	0.94, 1.54
Eczema	24.8%	23.6%	0.95	0.71, 1.28	0.98	0.73, 1.33	26.3%	1.06	0.87, 1.30	1.08	0.88, 1.33	25.1%	1.01	0.90, 1.14	1.03	0.92, 1.16
Food allergies	4.3%	5.3%	1.23	0.65, 2.32	1.07	0.53, 2.15	3.9%	0.91	0.55, 1.50	0.87	0.51, 1.46	4.2%	0.98	0.71, 1.34	0.98	0.71, 1.34
Allergic rhinitis	18.9%	26.7%	1.41	1.08, 1.84	1.29	0.96, 1.74	18.4%	0.97	0.75, 1.26	0.95	0.73, 1.22	20.9%	1.10	0.96, 1.26	1.09	0.95, 1.24
**Puberty signs**																
Period	6.5%	4.1%	0.63	0.24, 1.68	0.46	0.15, 1.43	6.4%	0.99	0.51, 1.93	0.94	0.46, 1.90	7.9%	1.20	0.82, 1.77	1.13	0.79, 1.63
Breast development	67.5%	70.3%	1.04	0.88, 1.23	0.95	0.81, 1.11	61.9%	0.92	0.80, 1.05	0.89	0.78, 1.02	67.4%	1.00	0.93, 1.07	0.98	0.92, 1.05
Precocious puberty^a^	4.9%	4.9%	1.00	0.54, 1.87	0.80	0.40, 1.60	5.4%	1.10	0.67, 1.80	1.01	0.62, 1.67	6.2%	1.27	0.95, 1.69	1.20	0.91, 1.60
**Vision and dental**																
Wears eyeglasses	44.7%	47.3%	1.06	0.88, 1.28	1.03	0.85, 1.25	54.7%	1.22	1.09, 1.38	1.23	1.09, 1.39	44.1%	0.99	0.92, 1.07	0.98	0.91, 1.06
Strabismus	1.3%	2.9%	2.20	1.05, 4.61	1.99	0.91, 4.39	2.3%	1.74	0.93, 3.26	1.67	0.85, 3.28	3.0%	2.27	1.42, 3.64	2.18	1.37, 3.47
Astigmatism	13.9%	14.6%	1.05	0.73, 1.51	1.05	0.72, 1.52	19.6%	1.41	1.06, 1.87	1.43	1.08, 1.89	13.1%	0.94	0.80, 1.11	0.95	0.80, 1.12
Hyperopia	14.6%	14.4%	0.98	0.69, 1.41	0.97	0.68, 1.39	19.9%	1.36	1.06, 1.73	1.39	1.09, 1.77	15.1%	1.04	0.88, 1.21	1.04	0.89, 1.22
Myopia	13.7%	18.6%	1.36	0.93, 1.97	1.34	0.92, 1.96	13.9%	1.02	0.72, 1.43	1.03	0.73, 1.44	13.2%	0.96	0.81, 1.16	0.95	0.80, 1.14
Malposition of teeth/jaw	30.8%	41.5%	1.35	1.09, 1.67	1.42	1.15, 1.75	27.9%	0.91	0.74, 1.11	0.95	0.78, 1.16	33.5%	1.09	0.99, 1.20	1.12	1.01, 1.23
**Behaviour and associated complaints**																
**SDQ score** (at risk)																
Emotional	21.6%	23.0%	1.06	0.79, 1.43	1.04	0.77, 1.41	19.3%	0.90	0.70, 1.14	0.88	0.69, 1.12	23.3%	1.08	0.95, 1.23	1.07	0.94, 1.22
Conduct	13.3%	12.6%	0.94	0.60, 1.47	0.84	0.54, 1.30	12.1%	0.91	0.68, 1.22	0.86	0.64, 1.16	14.6%	1.10	0.92, 1.31	1.04	0.87, 1.23
Hyperactivity	13.7%	18.2%	1.33	0.94, 1.87	1.27	0.89, 1.79	13.9%	1.01	0.76, 1.34	0.94	0.71, 1.25	15.8%	1.15	0.98, 1.35	1.09	0.92, 1.27
Peer relations	11.9%	16.5%	1.38	0.89, 2.14	1.31	0.87, 1.97	14.5%	1.21	0.89, 1.66	1.13	0.84, 1.54	11.1%	0.93	0.77, 1.14	0.90	0.74, 1.10
Global score	11.0%	12.8%	1.15	0.74, 1.78	1.07	0.69, 1.66	13.3%	1.21	0.88, 1.66	1.13	0.83, 1.54	13.6%	1.23	1.02, 1.48	1.16	0.97, 1.40
**Physical complaints**																
Abdominal pain	50.5%	49.0%	0.97	0.81, 1.16	0.98	0.82, 1.18	45.9%	0.91	0.79, 1.04	0.92	0.80, 1.06	48.1%	0.95	0.89, 1.02	0.95	0.89, 1.02
Constipation^b^	11.5%	15.8%	1.37	0.95, 1.99	1.34	0.89, 2.04	9.4%	0.82	0.59, 1.15	0.80	0.56, 1.13	11.8%	1.03	0.85, 1.25	1.00	0.82, 1.22
Headaches	20.8%	19.5%	0.94	0.68, 1.28	0.96	0.69, 1.33	22.4%	1.07	0.85, 1.35	1.06	0.84, 1.34	22.3%	1.07	0.94, 1.22	1.07	0.94, 1.21
**Sleep**																
Duration (< 9 h/> 12 h)	2.7%	0.9%	0.35	0.13, 0.94	0.32	0.11, 0.94	1.6%	0.58	0.25, 1.35	0.59	0.24, 1.41	3.0%	1.11	0.74, 1.68	1.09	0.72, 1.65
Lack (often)	13.9%	12.6%	0.90	0.57, 1.44	0.90	0.56, 1.46	13.3%	0.96	0.69, 1.33	0.95	0.69, 1.30	15.4%	1.11	0.94, 1.31	1.09	0.93, 1.29
Difficulty falling asleep (often)	22.8%	16.0%	0.72	0.51, 1.01	0.72	0.51, 1.01	20.3%	1.04	0.85, 1.26	1.02	0.84, 1.24	15.3%	1.03	0.93, 1.15	1.03	0.92, 1.14

*Note:* The quasi Poisson regression was performed for binomial outcomes. All the binomial outcomes had categories yes/no and reference category was set as “no”, unless stated otherwise. Analyses adjusted for: mother's age, country of birth, education level, employment status; household income, CSP; mother's history of diabetes mellitus, arterial hypertension, infertility treatment, pre‐pregnancy BMI, smoking during pregnancy; foetal growth restriction, child's sex.

^a^ Precocious puberty is defined as an appearance of pubic hair before the age of 9.5 for boys and 8 years for girls, or breasts before the age of 8, or period before the age of 11.

^b^ Defined as ≤ 2 stools per week.

#### 3.2.2 Puberty

No differences in the prevalence of thelarche or menarche were observed across GA groups (Table 2). However, the association between pubarche by age 10 and GA appeared to follow a dose–response pattern, with the highest odds observed among MPT: aOR 1.97 (95% CI 1.29–3.01) (Table 4).

#### 3.2.3 Motor and Cognitive

The risk of poor performance in one‐leg exercises was 63% higher among MPT (95% CI 0.81, 3.32) and 80% among LPT (95% CI 1.14, 2.82), compared to FT (Table 3). Concerning cognition, only MPT scored on average 0.64 (95% CI −1.17, −0.11) points lower on matrix, and 0.74 (95% CI −1.23, −0.26) points lower on puzzle subtests of the WISC‐V [35, 36], compared to FT.

**TABLE 3:** Descriptive analysis (prevalence/mean (SD)) and outcome‐wide regression results of measured outcomes from the weighted home visit subsample, *n* = 6418.

Outcomes	Gestational age															
	39–40 GA, *n* = 4250	32–33 GA, *n* = 231					34–36 GA, *n* = 496					37–38 GA, *n* = 1441				
			Unadjusted		Adjusted			Unadjusted		Adjusted			Unadjusted		Adjusted	
	Mean (SD)	Mean (SD)	β	95% CI	β	95% CI	Mean (SD)	β	95% CI	β	95% CI	Mean (SD)	β	95% CI	β	95% CI
**Anthropometry**	(1.00)															
Waist/Height (WH)	0.4 (0.1)	0.5 (0.1)	0.00	−0.01, 0.02	0.00	−0.01, 0.01	0.5 (0.1)	0.00	0.00, 0.01	0.00	−0.01, 0.01	0.5 (0.1)	0.01	0.00, 0.01	0.00	0.00, 0.01
WH ratio *z* score	0.1 (1.1)	0.2 (1.1)	0.09	−0.13, 0.31	0.08	−0.15, 0.31	0.2 (1.1)	0.09	−0.06, 0.25	0.03	−0.13, 0.18	0.2 (1.1)	0.14	0.05, 0.23	0.08	−0.01, 0.16
Weight/age *z*‐score	0.3(1.0)	0.2(1.1)	−0.15	−0.36, 0.05	−0.14	−0.35, 0.06	0.3(1.2)	0.00	−0.16, 0.15	−0.03	−0.20, 0.13	0.4(1.0)	0.13	0.04, 0.21	0.09	0.01, 0.17
Height/age *z*‐score	0.3 (1.1)	0.1 (1.0)	−0.27	−0.45, −0.10	−0.27	−0.45, −0.10	0.3 (1.0)	−0.01	−0.17, 0.14	−0.03	−0.19, 0.13	0.4 (1.1)	0.07	−0.02, 0.15	0.06	−0.02, 0.15
BMI/age *z*‐score	0.3 (1.2)	0.2 (1.3)	−0.05	−0.32, 0.21	−0.04	−0.30, 0.22	0.3 (1.2)	0.01	−0.18, 0.19	−0.03	−0.21, 0.16	0.4 (1.2)	0.15	0.05, 0.25	0.10	0.00, 0.19
**BP percentiles** (mmHg)																
Systolic	31.6 (27.6)	31.9 (27.2)	0.26	−4.37, 4.90	−0.67	−5.52, 4.19	32.3 (27.8)	0.74	−3.23, 4.72	−0.26	−4.13, 3.60	32.7 (29.0)	1.13	−1.17, 3.42	0.37	−1.87, 2.60
Diastolic	51.3 (23.4)	51.2 (21.6)	−0.03	−3.92, 3.87	−0.30	−3.96, 3.36	51.4 (21.8)	0.19	−3.15, 3.53	−0.87	−4.33, 2.60	52.4 (23.2)	1.12	−0.74, 2.98	0.72	−1.12, 2.55
HR (beats/min)	74.7 (10.6)	75.6 (10.9)	0.86	−1.25, 2.98	0.84	−1.26, 2.93	75.6 (10.9)	0.91	−0.79, 2.61	0.66	−1.00, 2.33	75.7 (10.8)	0.98	0.13, 1.83	0.96	0.15, 1.78
**Physical fitness**																
Jump length (m)	1.2 (0.2)	1.2 (0.2)	−0.01	−0.07, 0.04	0.01	−0.04, 0.06	1.2 (0.2)	−0.06	−0.09, −0.02	−0.04	−0.07, −0.01	1.2 (0.2)	0.00	−0.02, 0.02	0.01	−0.01, 0.03
*N* of sit‐ups	11.6 (4.3)	11.6 (4.4)	−0.02	−0.88, 0.84	0.00	−0.89, 0.89	11.1 (4.7)	−0.49	−1.23, 0.26	−0.39	−1.09, 0.32	11.5 (4.6)	−0.11	−0.48, 0.26	0.02	−0.33, 0.37
**Cognitive/intelligence**																
Matrix score ^a^	10.0 (2.5)	9.1 (2.7)	−0.84	−1.36, −0.32	−0.64	−1.17, −0.11	9.8 (2.4)	−0.13	−0.48, 0.22	0.08	−0.25, 0.42	9.8 (2.6)	−0.16	−0.37, 0.06	−0.04	−0.24, 0.16
Puzzle score ^a^	10.5 (2.5)	9.6 (2.6)	−0.90	−1.46, −0.35	−0.74	−1.23, −0.26	10.2 (2.3)	−0.32	−0.67, 0.02	−0.06	−0.39, 0.27	10.3 (2.4)	−0.24	−0.44, −0.04	−0.13	−0.32, 0.06
PPVT score ^b^	98.0 (12.8)	95.2 (14.5)	−2.82	−5.60, −0.05	−2.24	−4.78, 0.29	96.1 (14.3)	−1.90	−4.10, 0.29	−0.58	−2.72, 1.56	97.1 (12.7)	−0.90	−1.93, 0.13	−0.16	−1.10, 0.77

	%	%	RR	95% CI	RR	95% CI	%	RR	95% CI	RR	95% CI	%	RR	95% CI	RR	95% CI
**Motor skills**																
Dribbling (0–1/4)	19.0%	15.6%	0.82	0.57, 1.17	0.72	0.48, 1.08	22.1%	1.16	0.86, 1.57	1.12	0.82, 1.52	21.7%	1.14	0.97, 1.34	1.12	0.96, 1.30
One leg (0–2/5)	5.5%	9.0%	1.62	0.76, 3.46	1.63	0.81, 3.32	10.6%	1.93	1.23, 3.01	1.80	1.14, 2.82	6.3%	1.13	0.83, 1.55	1.11	0.82, 1.51
Throwing a ball (0–1/4)	23.8%	22.8%	0.96	0.67, 1.38	0.88	0.61, 1.27	28.0%	1.18	0.90, 1.53	1.07	0.82, 1.41	25.1%	1.06	0.92, 1.21	1.03	0.90, 1.18
Jumping (0–1/4)	10.8%	11.4%	1.05	0.65, 1.70	1.00	0.58, 1.72	13.3%	1.24	0.84, 1.83	1.11	0.73, 1.67	14.6%	1.36	1.09, 1.69	1.32	1.06, 1.64
Global score (0–8/17)	3.5%	4.4%	1.25	0.64, 2.43	1.18	0.55, 2.55	6.0%	1.71	0.91, 3.23	1.54	0.81, 2.94	4.9%	1.40	0.94, 2.09	1.31	0.88, 1.96

*Note:* Linear regression was performed for continuous outcomes (β, 95% CI), quasi Poisson regression for binomial outcomes (RR, 95% CI). Analyses adjusted for: mother's age, country of birth, education level, employment status; household income, CSP; mother's history of diabetes mellitus, arterial hypertension, infertility treatment, pre‐pregnancy BMI, smoking during pregnancy; foetal growth restriction, child's sex.

^a^ Standard score (mean = 10, SD = 3) from the matrix subtest of Fluid Reasoning Index (FRI) and puzzle subtest of the Visual Spatial Index (VSI) of the WISC‐V.

^b^ Peabody Picture Vocabulary Test (PPVT) 5, adapted to French, assessing children's knowledge of spoken words and receptive vocabulary.

**TABLE 4:** Descriptive analysis (prevalence) and outcome‐wide regression results of reported outcomes from the weighted telephone interview subsample, *n* = 8372.

OUTCOMES	GESTATIONAL AGE															
	39–40 GA, *n*= 5466	32–33 GA, *n* = 328					34–36 GA, *n* = 680					37–38 GA, *n* = 1898				
			Unadjusted		Adjusted			Unadjusted		Adjusted			Unadjusted		Adjusted	
	%	%	OR	95% CI	OR	95% CI	%	OR	95% CI	OR	95% CI	%	OR	95% CI	OR	95% CI
**Pubic hair** (ref no)	(1.00)															
Yes	28.3%	41.1%	1.80	1.23, 2.64	1.97	1.29, 3.01	35.3%	1.39	1.03, 1.86	1.49	1.08, 2.05	30.7%	1.10	0.94, 1.28	1.13	0.96, 1.33
Don't know	16.5%	15.3%	1.18	0.72, 1.92	1.14	0.70, 1.87	16.2%	1.14	0.78, 1.67	1.18	0.81, 1.74	15.9%	1.02	0.83, 1.26	0.98	0.80, 1.21
**Behavioural difficulties SDQ** (ref normal)																
Emotional																
Borderline	11.6%	11.8%	1.05	0.69, 1.58	0.94	0.59, 1.50	13.7%	1.19	0.82, 1.73	1.15	0.78, 1.70	10.8%	0.96	0.78, 1.17	0.95	0.77, 1.16
At risk	21.5%	23.2%	1.08	0.74, 1.57	1.05	0.70, 1.57	20.1%	0.93	0.69, 1.26	0.91	0.67, 1.24	24.3%	1.17	0.99, 1.37	1.15	0.97, 1.35
Conduct																
Borderline	13.4%	16.6%	1.29	0.76, 2.17	1.27	0.73, 2.24	11.4%	0.82	0.57, 1.19	0.81	0.56, 1.18	11.4%	0.84	0.69, 1.03	0.82	0.67, 1.01
At risk	13.5%	12.5%	0.93	0.59, 1.46	0.83	0.51, 1.35	13.7%	0.99	0.72, 1.36	0.94	0.67, 1.32	14.8%	1.09	0.89, 1.33	1.02	0.84, 1.25
Hyperactivity																
Borderline	7.3%	8.2%	1.17	0.71, 1.94	1.20	0.68, 2.14	7.3%	1.00	0.62, 1.62	0.93	0.57, 1.52	8.2%	1.16	0.91, 1.48	1.14	0.89, 1.46
At risk	14.1%	18.1%	1.39	0.91, 2.12	1.33	0.86, 2.07	14.5%	1.04	0.75, 1.43	0.97	0.69, 1.36	15.9%	1.17	0.98, 1.41	1.11	0.92, 1.34
Peer relations																
Borderline	10.5%	10.9%	1.12	0.68, 1.84	0.96	0.55, 1.67	12.0%	1.21	0.81, 1.81	1.10	0.74, 1.66	11.0%	1.06	0.84, 1.33	1.01	0.80, 1.27
At risk	12.1%	16.5%	1.43	0.87, 2.33	1.32	0.82, 2.13	14.5%	1.26	0.88, 1.81	1.18	0.81, 1.71	11.7%	0.97	0.78, 1.20	0.92	0.73, 1.14
Global score																
Borderline	9.6%	10.6%	1.14	0.72, 1.79	0.99	0.60, 1.61	7.5%	0.78	0.50, 1.22	0.75	0.47, 1.19	11.0%	1.21	0.97, 1.51	1.14	0.91, 1.43
At risk	11.3%	12.7%	1.12	0.72, 1.73	1.04	0.64, 1.69	13.8%	1.21	0.85, 1.73	1.13	0.79, 1.62	13.8%	1.27	1.04, 1.57	1.20	0.97, 1.48

*Note:* Multinomial logistic regression was performed for non‐binomial categorical outcomes. Analyses adjusted for: mother's age, country of birth, education level, employment status; household income, CSP; mother's history of diabetes mellitus, arterial hypertension, infertility treatment, pre‐pregnancy BMI, smoking during pregnancy; foetal growth restriction, child's sex.

**TABLE 5:** Descriptive analysis (prevalence) and outcome‐wide regression results of measured outcomes from the weighted home visit subsample, *n* = 6418.

Outcomes	Gestational age															
	39–40 GA, *n* = 4250	32–33 GA, *n*= 231					34–36 GA, *n* = 496					37–38 GA, *n*= 1441				
			Unadjusted		Adjusted			Unadjusted		Adjusted			Unadjusted		Adjusted	
	%	%	OR	95% CI	OR	95% CI	%	OR	95% CI	OR	95% CI	%	OR	95% CI	OR	95% CI
**Anthropometry**	(1.00)															
WH ratio *z* score (ref 0)																
−2SD	0.1%	0.4%	4.60	0.50 23.27	2.66	0.23, 30.9	0.3%	2.54	0.30, 21.4	2.73	0.57, 13.1	0.2%	1.71	0.64, 4.58	1.73	0.58, 5.14
2SD	6.3%	8.4%	1.37	0.75, 2.51	1.18	0.54, 2.56	7.4%	1.20	0.67, 2.14	0.93	0.46, 1.86	8.3%	1.34	0.98, 1.82	1.15	0.83, 1.59
Weight/age *z*‐score (ref 0)																
< −1SD	9.3%	14.0%	1.54	0.87, 2.71	1.07	0.57, 2.01	11.0%	1.23	0.76, 1.99	0.90	0.55, 1.47	8.9%	0.99	0.75, 1.29	0.89	0.67, 1.19
[1 SD, 2 SD]	19.6%	17.4%	0.90	0.52, 1.57	0.78	0.43, 1.41	23.2%	1.22	0.84, 1.78	1.06	0.73, 1.53	18.6%	0.97	0.80, 1.18	0.91	0.74, 1.13
≥ 2 SD	6.3%	5.2%	0.84	0.38, 1.87	0.66	0.25, 1.76	2.8%	0.46	0.27, 0.81	0.32	0.16, 0.63	9.2%	1.49	1.09, 2.03	1.27	0.91, 1.76
Height/age *z*‐score (ref 0)																
< −1 SD	9.2%	11.8%	1.09	0.66, 1.78	0.99	0.58, 1.70	9.4%	0.96	0.57, 1.61	0.90	0.53, 1.53	10.3%	1.06	0.81, 1.40	1.03	0.78, 1.36
[1 SD, 2 SD]	19.3%	14.2%	0.63	0.36, 1.10	0.61	0.33, 1.12	20.2%	1.02	0.70, 1.49	0.97	0.68, 1.39	18.0%	0.93	0.75, 1.14	0.93	0.76, 1.14
≥ 2 SD	6.6%	3.2%	0.44	0.19, 1.03	0.38	0.15, 0.98	5.4%	0.80	0.43, 1.48	0.65	0.33, 1.29	7.7%	1.15	0.85, 1.57	1.11	0.81, 1.52
BMI/age *z*‐score (ref 0)																
< −1 SD	13.5%	19.4%	1.45	0.85, 2.46	1.24	0.70, 2.20	16.7%	1.25	0.83, 1.89	1.12	0.73, 1.72	10.9%	0.74	0.58, 0.95	0.75	0.59, 0.96
[1 SD, 2 SD]	17.7%	17.6%	1.03	0.60, 1.76	0.96	0.54, 1.72	23.0%	1.37	0.91, 2.06	1.31	0.87, 1.96	18.7%	1.00	0.81, 1.23	0.95	0.76, 1.18
≥ 2 SD	8.9%	9.2%	1.14	0.61, 2.21	0.99	0.43, 2.26	6.7%	0.82	0.47, 1.43	0.62	0.33, 1.19	10.9%	1.22	0.92, 1.63	1.05	0.78, 1.40
IOTF BMI (ref normal weight) (ref Normal weight)																
Underweight	9.5%	15.9%	1.57	0.91, 2.73	1.32	0.73, 2.39	12.5%	1.25	0.77, 2.04	1.06	0.65, 1.73	7.8%	0.74	0.56, 0.99	0.76	0.57, 1.00
Overweight	14.9%	11.6%	0.79	0.45, 1.39	0.70	0.35, 1.39	19.5%	1.34	0.88, 2.03	1.18	0.76, 1.82	15.3%	0.99	0.79, 1.25	0.92	0.73, 1.17
Obesity	3.6%	4.4%	1.33	0.54, 3.25	0.98	0.27, 3.59	1.6%	0.49	0.25, 0.98	0.33	0.13, 0.80	6.3%	1.80	1.21, 2.67	1.59	1.06, 2.37
**BP level** ^a^ (ref normal)																
Pre‐elevated	4.3%	3.1%	0.66	0.23, 1.88	0.76	0.28, 2.10	3.1%	0.71	0.40, 1.27	0.73	0.40, 1.34	5.1%	1.19	0.82, 1.73	1.10	0.76, 1.61
Elevated	4.2%	4.0%	0.87	0.42, 1.81	0.96	0.42, 2.24	6.2%	1.35	0.71, 2.59	1.31	0.68, 2.52	6.0%	1.35	0.93, 1.95	1.31	0.90, 1.89

*Note:* Multinomial logistic regression was performed for non‐binomial categorical outcomes. Analyses adjusted for: mother's age, country of birth, education level, employment status; household income, CSP; mother's history of diabetes mellitus, arterial hypertension, infertility treatment, pre‐pregnancy BMI, smoking during pregnancy; foetal growth restriction, child's sex.

^a^ BP level was defined using the American Academy of Paediatrics cut‐offs.

**TABLE 6:** Sensitivity analysis for unmeasured confounding (*E*‐values) conducted in telephone interview and home visit subsamples for outcome‐wide modified Poisson, linear, and logistic regressions.

Outcomes	Gestational age					
	32–33 GA		34–36 GA		37–38 GA	
	*E*‐value	*E*‐value for CI	*E*‐value	*E*‐value for CI	*E*‐value	*E*‐value for CI
**Respiratory and allergies**						
Allergic rhinitis	1.90	1				
**Visual and dental**						
Wears eyeglasses			1.76	1.40		
Strabismus	3.39	1	2.87	1	3.78	2.08
Astigmatism			2.21	1.37		
Hypermetropia			2.13	1.40		
Myopia	2.01	1				
Malposition of teeth/jaw	2.19	1.57			1.49	1.10
**Sleep duration**	5.16	1.32				
**Anthropometry**						
Height for age *z*‐score (continuous)	1.85	1.39				
**Cognitive tests**						
Matrix score standard	1.84	1.25				
Puzzle score standard	1.98	1.44				
**Exercises (motor skills)**						
One leg (0–2/5)	2.64	1	3.00	1.54		
**Pubic hair** (yes)	2.16	1.53	1.74	1.24		
**Anthropometry**						
Height for age *z*‐score (ref 0)						
≥ 2 SD	4.70	1.16				
IOTF BMI (ref normal weight)						
Obesity			5.51	1.81	2.56	1.31

*Note:* *E*‐values in grey: confidence interval crosses 1, so the value of *E*‐value for confidence interval is 1.

*E*‐value calculation method was adapted to the type of regression conducted.

#### 3.2.4 Dental Malposition and Anthropometry

MPT had around 15%–75% higher risk of teeth or jaw malposition, compared to FT peers (Table 2). They also had on average 0.45 to 0.10 units lower height‐for‐age z‐score (Table 3)—multinomial logistic regression showed their odds of being tall at/above 2 z‐scores for height‐for‐age were 62% lower, compared to FT (Table 5).

#### 3.2.5 Other Results

MPT had around 30% higher risk of allergic rhinitis compared to FT peers (Table 2).

Among the outcomes known to be associated with earlier preterm births, we note a modest increase in the prevalence of asthma in all our GA groups, compared to FT, as well as being classified as ‘at risk’ on the SDQ hyperactivity and peer relations scales among MPT.

Except for cognitive, BP, and, to a lesser degree, behavioural outcomes, adjustment for confounding had a negligible effect on the strength of the associations.

### 3.3 Sensitivity Analysis

The *E*‐value of 3.78 implies that a confounder, or a set of confounders, would need to be associated with at least a 3.78‐fold increase in the risk of strabismus and 3.78 times more prevalent among ET than FT to explain the observed RR (Table 6).

### 3.4 Other Analyses

The supplemental analyses, replicated in the additional adjustment and singleton subsamples, slightly shifted some effect estimates and confidence intervals (Appendices, Tables S2–S5). However, these changes did not alter overall conclusions. It's worth noting that we observed increased risk of hyperactivity among MPT, compared to FT, in the singleton subsample.

## 4 Comment

### 4.1 Principal Findings

We observed no major difference between the GA groups for respiratory, anthropometric, and cardiometabolic outcomes. Nonetheless, a 2‐fold increase in risk for strabismus was observed among preterm and ET children at age 10 (prevalence around 3%), as well as a 40% increased risk of hyperopia and astigmatism among LPT. MPT and LPT exhibited poorer balance skills and a higher prevalence of pubarche at age 10, compared to FT peers. Additionally, MPT, but not the other two groups, had increased risk of allergic rhinitis, myopia, dental malposition, as well as a lower average WISC‐V [35] matrix and puzzle standardised scores.

### 4.2 Strengths of the Study

This study has several strengths. It is based on data from two large cohorts, including between 6400 and 8300 participants. The risk of bias due to death as a competing event is low, as only 0.1% of our study population died between birth and age 10. To address selection bias due to attrition and enhance external validity and generalisability, we applied sampling and non‐inclusion/participation weights. Missing data from partial non‐response were limited and handled using a robust multiple imputations strategy, following expert recommendations. Pre‐exposure confounders were carefully selected based on a literature review and identification of a valid adjustment set using DAGitty. We employed outcome‐wide analyses, which minimise reporting bias and allow for the comparison of the strength of associations within the same population using the same analytic method. Both adjusted and unadjusted estimates were reported for transparency: adjusted estimates isolate the effect of GA from related factors, while unadjusted estimates offer predictive insights that may be relevant from a clinical perspective. Finally, we conducted sensitivity analyses to assess the robustness of our findings to unmeasured confounding.

### 4.3 Limitations of the Data

Some outcomes were parent‐reported, introducing potential information bias; however, the absence of a clear dose–response pattern across exposure groups reduces this concern. The attrition between inclusion and the 10‐year follow‐up may have introduced selection bias; although it was non‐differential by GA. The outcome‐wide analytic approach limits the ideal confounding adjustment for individual associations. Lastly, due to heterogeneous subgroup sample sizes, we did not perform corrections for multiple testing.

### 4.4 Interpretation

#### 4.4.1 Vision

Strabismus results from ocular muscle imbalance or neurologic disorders like cerebral palsy [53, 54], while refractive error (hyperopia, myopia, astigmatism) stems from mismatches between the retina and the eye's optical focal plane [55]. A strabismus prevalence of 8.3% at age 5.5 years was previously reported for children born at 32–34 GA who underwent clinical examination in the EPIPAGE 2 cohort [56]. Prior publications found an increased risk of strabismus and refractive error for preterm (< 37 GA) born children [57, 58]. Our study extends those findings to MPT, LPT, and ET populations and builds on them by identifying specific risks linked to each GA group. There are numerous hypotheses behind visual impairments in preterm children [59], one of which is the exposure of immature eye structures to early visual stimuli [60]. Based on this mechanism, we expected a higher refractive error prevalence in MPT compared to LPT and ET, but our findings do not fully support this expectation. Our descriptive analysis shows that a higher proportion of LPT and ET mothers smoked during pregnancy—revealing another potential cause of the higher prevalence of refractive error among LPT and strabismus among ET [61]. While the higher rate of eyeglasses use in our LPT sample might suggest surveillance bias, we cannot rule out reporting bias, as our estimates of visual impairments rely on parental reports. Nonetheless, the cost–benefit of a targeted ophthalmologic screening for these children should be explored.

#### 4.4.2 Puberty

The prevalence of pubarche at age 10 was higher among MPT and LPT born boys and girls, compared to those born FT. The identified odds likely overestimate the risks, as the prevalence of pubic hair appearance ranged from 35.3% to 41.1% in these subgroups. It is unclear whether the increased prevalence reflects early pubarche in those children, given that pubarche at age 10 falls within the physiological timeframe [62]. However, preterm birth and associated conditions—including foetal growth restriction and small for gestational age—have been linked to elevated dehydroepiandrosterone sulphate (DHEA‐S) levels, which may contribute to earlier pubarche onset [63, 64]. A systematic review reported inconsistent findings on puberty onset in preterm children [65], underscoring the need for further investigation.

#### 4.4.3 Motor and Cognitive

We observed poorer balance in children born MPT and LPT, demonstrated by weaker one‐leg exercise performance compared to FT peers. Similarly, 7–8‐year‐old MPT/LPT children showed impaired heel‐to‐toe balance and manual dexterity [14], with increased risks of motor impairment reported up to the age of 16 [15]. GA has also been linked to a higher risk of suspected developmental coordination disorder in very preterm and MPT populations aged 3–10 [66]; our results suggest this should be further explored in LPT populations. These findings may be explained via reduced cerebellar white matter volume observed in adolescents born preterm, with more pronounced reductions linked to lower GA [67].

Matrix and puzzle standardised scores from the WISC‐V intelligence scale were on average lower for MPT children compared to FT peers, but not for the other two groups. The matrix subtest assesses problem‐solving, while the puzzle subtest evaluates non‐verbal reasoning and attention to detail [36]. Lower average WISC‐V full scale IQ scores have been reported for MPT/LPT in childhood [18] and LPT in childhood and adulthood [16, 17]. We found no significant difference in matrix and puzzle scores between LPT and FT children at age 10, possibly due to limited comparability with studies using the full WISC‐V to assess full‐scale, processing, and verbal IQ, whereas we assessed only two subtests. Nonetheless, our findings on poorer problem‐solving skills partially align with earlier research reporting reduced spatial memory efficiency associated with problem‐solving tasks across all preterm birth groups in adulthood [68]. Our study observed this difference only among MPT, whereas LPT and ET children did not differ from their FT peers. It remains unclear whether the observed WISC‐V differences have clinical or practical relevance. We also observed a lower average PPVT‐5 [37] score among MPT, compared to FT counterparts, but this association got weaker with adjustment.

#### 4.4.4 Malposition of Teeth or Jaw

Our results showed an increased risk of teeth or jaw malposition among MPT and no significant difference for LPT and ET compared to their FT peers. Differences in craniofacial morphology between extremely and very preterm children and their term peers at 8–10 years were previously reported, including a shorter anterior cranial base, shorter maxilla, and more retroclined and retruded lower incisors [69]. Additionally, some alterations in dental and oral development have been reported for preterm children, including palatal and cleft anomalies [70]. Our study offers new insights into these long‐term outcomes in MPT children. While the exact mechanisms remain unclear, oral trauma from intubation or other vital interventions at birth may mediate these associations.

#### 4.4.5 Anthropometry

Our findings suggest that only MPT are shorter than their FT peers. A previous study reported an inverse association between GA and mean weight and height from birth to early adolescence, with minimal but observable differences in weight and height trajectories between LPT and FT peers between ages 7 and 14 [71]. This aligns with our findings, as differences in height between preterm and term children were present but negligible. In replicated singleton analyses, anthropometric associations were attenuated, suggesting that size differences are partly explained by twin‐singleton differences.

### 4.5 Results That Merit Further Investigation

We observed a higher prevalence of several outcomes among preterm and ET children compared to their FT peers, although these differences were not large enough to ascertain increased risks across the GA groups. Notably, asthma was more prevalent among preterm and ET children, while hyperactivity and relational behavioural issues were more prevalent among MPT. Previous studies have consistently shown an increased risk of obstructive airway disease across preterm birth groups [72, 73, 74, 75]. We emphasise the importance of continued monitoring of these risks among MPT, LPT, and ET populations, especially given the limitations of outcome‐wide analysis.

## 5 Conclusions

In conclusion, this study concurrently assessed multiple long‐term health and developmental outcomes in MPT, LPT, and ET born children in comparison to their FT peers. It found no major differences for most assessed outcomes, including respiratory, anthropometric, and cardiometabolic. However, it identified an increased risk of visual impairments across GA groups, dental impairments in MPT, and confirmed previously reported inverse associations between GA and motor and intellectual difficulties.

## Author Contributions

The authors take full responsibility for this article.

## Ethics Statement

As required by French law and regulations, the National Committee on Information Processing in Health Research (*Comité Consultatif sur le Traitement de l'information en matière de Recherche dans le Domaine de la Santé*), the National Data Protection Authority (*Commission Nationale Informatique et Liberté*), and the Committee for Protection of Persons Engaged in Research (*Comité de Protection des Personnes*) approved both cohorts.

## Consent

ELFE: Informed consent for participation was signed at birth by the parents or the mother alone, with the father informed of his right to deny consent. EPIPAGE 2: Data collection occurred after families had received information and agreed to participate in the study. At the 10‐year follow‐up for both cohorts, one parent signed an informed consent and attested that the second parent had been informed about the child's participation, while children were asked for an oral consent.

## Conflicts of Interest

The authors declare no conflicts of interest.

## Supporting information


**Data S1:** ppe70069‐sup‐0001‐Supinfo01.zip.

## Data Availability

The data supporting the findings cannot be freely shared due to ethical and legal constraints. The numerous variables included in this study could potentially, when combined, re‐identify anonymized participants through specific characteristics, thereby compromising personal data. Consequently, the French ethics authority prohibits the unrestricted release of these data. However, access can be granted upon request to the principal investigators. Interested parties may contact contact@elfe-france.fr for the ELFE cohort and accesdonnees.epipage@inserm.fr or by using information provided on the data access website: https://epipage2.inserm.fr/index.php/en/related‐research/265‐dataaccess‐and‐questionnaires for the EPIPAGE 2 cohort.

## Endnotes

^1^
While the targeted age at follow‐up was 10.5 years, we will use 10 years throughout the article to enhance readability.

^2^
Note that these subsamples largely overlap, as shown in Figure 1. (6302 participants in common).
