# Supplementary material for: Health and Development of Children Born Moderate and Late Preterm and Early Term at Age 10 in French Birth Cohorts ELFE and EPIPAGE 2
Source: Paediatr Perinat Epidemiol. 2025 Sep 29;40(1):34–52. doi: 10.1111/ppe.70069 (PMC12853227; doi:10.1111/ppe.70069)
Supplement: Supplementary file 1 — Data S1: ppe70069‐sup‐0001‐Supinfo01.zip. [file PPE-40-34-s001.zip › Appendix 2 percentage missing.docx]

**Appendix 2**

The list of missing data for the outcomes assessed in the study (before multiple imputations)

| **Telephone interview subsample** | | **Home visit subsample** | |
| --- | --- | --- | --- |
| **Variables** | **Missing** | **Variables** | **Missing** |
| **Respiratory and allergies** |  | **Anthropometry** |  |
| Current asthma MEDALL definition | 1.1% (110) | Waist-height ratio | 3.1% (242) |
| Eczema | 1.3% (131) | Weight/age z-score | 0.1% (6) |
| Food allergies | 1.8% (180) | Height/age z-score | 0.2% (12) |
| Allergic rhinitis | 1.3% (131) | BMI/age z-score | 0.2% (12) |
| **Puberty signs** |  | IOTF BMI | 3.2% (250) |
| Period, ever | 1.4% (70) | **BP percentiles** |  |
| If no period, breast development | 6.5% (326) | Systolic | 4.3% (335) |
| Pubic hair (girls) | 1.3% (66) | Diastolic | 4.3% (335) |
| Pubic hair (boys) | 1.0% (54) | Heart rate | 4.3% (335) |
| **Vision and dental** |  | **Physical fitness** |  |
| Wears eyeglasses | 1.4% (140) | Jump length in cm | 5.9% (461) |
| Strabismus | 1.4% (141) | N of sit-ups | 5.0% (392) |
| Astigmatism | 1.4% (141) | **Cognitive functioning** |  |
| Hyperopia | 1.4% (141) | Matrix FSIQ score | 5.1% (400) |
| Myopia | 1.4% (141) | Puzzle PIQ score | 5.1% (402) |
| Malposition of the teeth/jaw | 1.4% (148) | PPVT score | 5.5% (430) |
| **Behavioural and associated complaints** |  | **Motor skills:** |  |
| Emotional SDQ | 1.6% (165) | Dribbling with a ball (0-1/4) | 3.6% (284) |
| Conduct SDQ | 1.6% (165) | One leg exercise (0-2/5) | 4.4% (346) |
| Hyperactivity SDQ | 1.6% (165) | Throwing a ball (0-1/4) | 3.8% (299) |
| Peer relations SDQ | 1.6% (165) | Jumping (0-1/4) | 6.5% (509) |
| Global Score SDQ | 1.6% (165) | Global score (0-8/17) | 7.3% (571) |
| **Physical complaints** |  |  |  |
| Abdominal pain | 1.5% (150) |  |  |
| Constipation | 1.4% (157) |  |  |
| Headache | 1.5% (149) |  |  |
| **Sleep** |  |  |  |
| Duration | 3.6% (371) |  |  |
| Lack | 1.6% (159) |  |  |
| Difficulty falling asleep | 1.6% (164) |  |  |
