# Supplementary material for: Health and Development of Children Born Moderate and Late Preterm and Early Term at Age 10 in French Birth Cohorts ELFE and EPIPAGE 2
Source: Paediatr Perinat Epidemiol. 2025 Sep 29;40(1):34–52. doi: 10.1111/ppe.70069 (PMC12853227; doi:10.1111/ppe.70069)
Supplement: Supplementary file 1 — Data S1: ppe70069‐sup‐0001‐Supinfo01.zip. [file PPE-40-34-s001.zip › Appendix 3 List of predictor variables.docx]

**Appendix 3**

List of the predictor variables for multiple imputations using miceRanger:

1. Maternal education level (for most variables) / Paternal education (father specific variables)
2. Maternal age (for most variables) / Paternal age (father specific variables)
3. Mother’s country of origin / Father’s country of origin (for father specific variables)
4. Maternal medical history of diabetes mellitus and gestational diabetes mellitus
5. Maternal medical history of arterial hypertension and gestational hypertension
6. Infertility treatment
7. Maternal smoking during pregnancy
8. Foetal growth restriction
9. Gestational age
10. Child’s sex

* miceRanger package allows for prediction matrices to be specified for each variable separately. 10 variables above were used as predictors for most of imputed variables, except for the highly correlated and dependent variables for which the sole predictors were the variables from which they were created (i.e., the sole predictor of the age in months at 10-year follow up is gestational age; the predictors for BMI-for-age z-score at 10 years are age, weight, and height).
