# Supplementary material for: Health and Development of Children Born Moderate and Late Preterm and Early Term at Age 10 in French Birth Cohorts ELFE and EPIPAGE 2
Source: Paediatr Perinat Epidemiol. 2025 Sep 29;40(1):34–52. doi: 10.1111/ppe.70069 (PMC12853227; doi:10.1111/ppe.70069)
Supplement: Supplementary file 1 — Data S1: ppe70069‐sup‐0001‐Supinfo01.zip. [file PPE-40-34-s001.zip › Appendix 4 Results of the analyses replicated in the singleton and ELFE subsamples.docx]

Appendix 4 Results of the analyses replicated in the singleton and ELFE subsamples

eTable 1 Descriptive analysis (prevalence) and outcome-wide regression results of reported outcomes from the weighted telephone interview subsample of singletons, N = 7891

| OUTCOMES | GESTATIONAL AGE | | | | | | | | | | | | | | | |
| --- | --- | --- | --- | --- | --- | --- | --- | --- | --- | --- | --- | --- | --- | --- | --- | --- |
|  | 39 – 40 GA | 32 – 33 GA, N = 202 | | | | | 34 – 36 GA, N = 496 | | | | | 37 – 38 GA, N = 1754 | | | | |
|  | N = 5439 |  | **Unadjusted** | | **Adjusted** | |  | **Unadjusted** | | **Adjusted** | |  | **Unadjusted** | | **Adjusted** | |
|  | % | **%** | **RR** | **95% CI** | **RR** | **95% CI** | **%** | **RR** | **95% CI** | **RR** | **95% CI** | **%** | **RR** | **95% CI** | **RR** | **95% CI** |
| **Respiratory and allergies** | (1.00) |  |  |  |  |  |  |  |  |  |  |  |  |  |  |  |
| Asthma | 6.2% | 8.2% | 1.31 | 0.70, 2.43 | 1.18 | 0.60, 2.32 | 8.4% | 1.34 | 0.82, 2.19 | 1.25 | 0.80, 1.95 | 7.6% | 1.21 | 0.94, 1.57 | 1.20 | 0.93, 1.55 |
| Eczema | 24.8% | 22.7% | 0.91 | 0.62, 1.35 | 0.97 | 0.65, 1.44 | 26.2% | 1.06 | 0.84, 1.33 | 1.08 | 0.86, 1.36 | 25.4% | 1.03 | 0.91, 1.15 | 1.05 | 0.93, 1.18 |
| Food allergies | 4.3% | 5.8% | 1.33 | 0.61, 2.88 | 1.24 | 0.53, 2.92 | 3.8% | 0.89 | 0.49, 1.62 | 0.86 | 0.46, 1.59 | 4.2% | 0.97 | 0.70, 1.35 | 0.98 | 0.71, 1.36 |
| Allergic rhinitis | 19.0% | 25.0% | 1.32 | 0.96, 1.82 | 1.19 | 0.83, 1.71 | 20.4% | 1.07 | 0.81, 1.43 | 1.04 | 0.78, 1.37 | 21.1% | 1.11 | 0.97, 1.28 | 1.10 | 0.96, 1.26 |
| **Puberty signs** |  |  |  |  |  |  |  |  |  |  |  |  |  |  |  |  |
| Period | 6.6% | 4.7% | 0.71 | 0.22, 2.33 | 0.45 | 0.12, 1.73 | 8.9% | 1.35 | 0.70, 2.64 | 1.29 | 0.64, 2.60 | 7.7% | 1.18 | 0.80, 1.75 | 1.12 | 0.77, 1.62 |
| Breast development | 67.8% | 73.3% | 1.08 | 0.88, 1.32 | 0.96 | 0.79, 1.17 | 63.5% | 0.94 | 0.80, 1.10 | 0.91 | 0.77, 1.06 | 68.2% | 1.01 | 0.94, 1.08 | 0.99 | 0.93, 1.06 |
| Precocious puberty ^a^ | 4.9% | 5.9% | 1.20 | 0.61, 2.38 | 0.92 | 0.43, 1.96 | 6.4% | 1.31 | 0.79, 2.17 | 1.28 | 0.77, 2.12 | 6.3% | 1.29 | 0.96, 1.73 | 1.23 | 0.93, 1.64 |
| **Vision and dental** |  |  |  |  |  |  |  |  |  |  |  |  |  |  |  |  |
| Wears eyeglasses | 44.7% | 44.5% | 0.99 | 0.78, 1.27 | 0.96 | 0.74, 1.24 | 53.3% | 1.19 | 1.04, 1.36 | 1.20 | 1.04, 1.37 | 44.1% | 0.99 | 0.91, 1.07 | 0.98 | 0.91, 1.06 |
| Strabismus | 1.3% | 3.3% | 2.56 | 1.13, 5.80 | 2.22 | 0.91, 5.43 | 2.0% | 1.51 | 0.69, 3.30 | 1.49 | 0.66, 3.39 | 3.0% | 2.31 | 1.42, 3.74 | 2.21 | 1.37, 3.57 |
| Astigmatism | 13.9% | 12.5% | 0.89 | 0.55, 1.44 | 0.92 | 0.57, 1.49 | 17.3% | 1.24 | 0.94, 1.64 | 1.27 | 0.96, 1.68 | 13.2% | 0.95 | 0.80, 1.12 | 0.95 | 0.81, 1.13 |
| Hyperopia | 14.6% | 14.0% | 0.96 | 0.61, 1.49 | 1.02 | 0.65, 1.59 | 21.2% | 1.45 | 1.11, 1.89 | 1.53 | 1.17, 1.99 | 14.9% | 1.02 | 0.87, 1.20 | 1.03 | 0.88, 1.22 |
| Myopia | 13.7% | 18.0% | 1.31 | 0.81, 2.10 | 1.25 | 0.79, 1.97 | 13.7% | 0.99 | 0.69, 1.42 | 0.96 | 0.67, 1.38 | 13.1% | 0.95 | 0.80, 1.14 | 0.93 | 0.78, 1.12 |
| Malposition of teeth/jaw | 30.8% | 40.4% | 1.31 | 1.00, 1.72 | 1.41 | 1.08, 1.85 | 27.9% | 0.90 | 0.73, 1.11 | 0.95 | 0.78, 1.17 | 33.1% | 1.07 | 0.97, 1.18 | 1.10 | 1.00, 1.22 |
| **Behaviour and associated complaints** |  |  |  |  |  |  |  |  |  |  |  |  |  |  |  |  |
| **SDQ score (At risk)** |  |  |  |  |  |  |  |  |  |  |  |  |  |  |  |  |
| Emotional | 21.6% | 24.7% | 1.14 | 0.79, 1.63 | 1.07 | 0.74, 1.54 | 19.3% | 0.89 | 0.68, 1.17 | 0.88 | 0.67, 1.15 | 24.2% | 1.12 | 0.99, 1.27 | 1.10 | 0.97, 1.25 |
| Conduct | 13.2% | 14.9% | 1.11 | 0.67, 1.84 | 0.92 | 0.56, 1.52 | 11.3% | 0.85 | 0.61, 1.19 | 0.80 | 0.57, 1.12 | 15.0% | 1.14 | 0.95, 1.36 | 1.07 | 0.89, 1.28 |
| Hyperactivity | 13.7% | 22.2% | 1.62 | 1.11, 2.38 | 1.51 | 1.03, 2.23 | 13.9% | 1.02 | 0.74, 1.41 | 0.95 | 0.69, 1.32 | 16.0% | 1.17 | 0.99, 1.38 | 1.11 | 0.94, 1.30 |
| Peer relations | 11.8% | 19.9% | 1.67 | 1.03, 2.73 | 1.46 | 0.93, 2.32 | 14.4% | 1.22 | 0.85, 1.74 | 1.11 | 0.79, 1.57 | 11.7% | 0.99 | 0.81, 1.21 | 0.95 | 0.78, 1.15 |
| Global score | 10.9% | 14.9% | 1.35 | 0.82, 2.21 | 1.16 | 0.70, 1.91 | 14.1% | 1.29 | 0.90, 1.83 | 1.20 | 0.86, 1.69 | 14.1% | 1.28 | 1.06, 1.55 | 1.21 | 1.00, 1.46 |
| **Physical complaints** |  |  |  |  |  |  |  |  |  |  |  |  |  |  |  |  |
| Abdominal pain | 50.4% | 48.2% | 0.95 | 0.76, 1.21 | 0.94 | 0.74, 1.19 | 49.3% | 0.98 | 0.85, 1.13 | 0.98 | 0.85, 1.13 | 49.2% | 0.97 | 0.91, 1.05 | 0.97 | 0.90, 1.04 |
| Constipation ^b^ | 11.5% | 17.2% | 1.48 | 0.96, 2.29 | 1.39 | 0.84, 2.31 | 8.8% | 0.77 | 0.51, 1.14 | 0.76 | 0.50, 1.15 | 12.0% | 1.04 | 0.85, 1.26 | 1.00 | 0.82, 1.22 |
| Headaches | 20.9% | 15.6% | 0.75 | 0.49, 1.14 | 0.74 | 0.48, 1.15 | 22.6% | 1.08 | 0.83, 1.41 | 1.07 | 0.82, 1.40 | 22.7% | 1.09 | 0.95, 1.24 | 1.09 | 0.95, 1.24 |
| **Sleep** |  |  |  |  |  |  |  |  |  |  |  |  |  |  |  |  |
| Duration (< 9h / >12h) | 2.7% | 1.0% | 0.36 | 0.10, 1.24 | 0.34 | 0.09, 1.31 | 2.0% | 0.74 | 0.31, 1.74 | 0.69 | 0.28, 1.70 | 3.2% | 1.17 | 0.77, 1.77 | 1.14 | 0.75, 1.73 |
| Lack (Often/Always) | 13.9% | 10.9% | 0.95 | 0.54, 1.66 | 0.93 | 0.52, 1.68 | 13.6% | 0.93 | 0.65, 1.34 | 0.93 | 0.65, 1.32 | 15.4% | 1.12 | 0.95, 1.33 | 1.11 | 0.94, 1.32 |
| Difficulty falling asleep (Always/Sometimes) | 22.8% | 18.1% | 0.77 | 0.51, 1.15 | 0.76 | 0.50, 1.14 | 20.2% | 0.96 | 0.76, 1.22 | 0.94 | 0.75, 1.18 | 23.7% | 1.03 | 0.92, 1.15 | 1.03 | 0.92, 1.15 |

The quasi Poisson regression was performed for binomial outcomes. All the binomial outcomes had categories yes/no and reference category was set as “no”, unless stated otherwise.

Analyses adjusted for: mother’s age, country of birth, education level, employment status; household income, and CSP; mother’s history of diabetes mellitus, arterial hypertension, infertility treatment, pre-pregnancy BMI, and smoking during pregnancy; foetal growth restriction, child’s sex

^a^ Precocious puberty is defined as an appearance of pubic hair before the age of 9.5 for boys and 8 years for girls, or breasts before the age of 8, or period before the age of 11. ^b^ Defined as ≤ 2 stools per week

eTable 2 Descriptive analysis (prevalence/mean (SD)) and outcome-wide regression results of measured outcomes from the weighted home visit subsample of singletons, N = 6064

| OUTCOMES | GESTATIONAL AGE | | | | | | | | | | | | | | | | | |
| --- | --- | --- | --- | --- | --- | --- | --- | --- | --- | --- | --- | --- | --- | --- | --- | --- | --- | --- |
|  | 39 – 40 GA | 32 – 33 GA, N = 136 | | | | | | 34 – 36 GA, N = 369 | | | | | 37 – 38 GA, N = 1334 | | | | | |
|  | N = 4225 |  | **Unadjusted** | | **Adjusted** | | |  | **Unadjusted** | | **Adjusted** | |  | **Unadjusted** | | **Adjusted** | | |
|  | **Mean (SD)** | **Mean (SD)** | **β** | **95% CI** | **β** | | **95% CI** | **Mean (SD)** | **β** | **95% CI** | **β** | **95% CI** | **Mean (SD)** | **β** | **95% CI** | **β** | **95% CI** | |
| **Anthropometry** | (1.00) |  |  |  |  | |  |  |  |  |  |  |  |  |  |  |  | |
| Waist/Height | 0.4 (0.1) | 0.4 (0.1) | 0.0 | -0.01, 0.02 | 0.0 | | -0.01, 0.01 | 0.5 (0.1) | 0.0 | -0.01, 0.01 | 0.0 | -0.01, 0.01 | 0.5 (0.1) | 0.0 | 0.00, 0.01 | 0.0 | 0.00, 0.01 | |
| WH ratio z score | 0.1 (1.1) | 0.1 (1.2) | 0.0 | -0.24, 0.32 | 0.0 | | -0.31, 0.31 | 0.2 (1.1) | 0.1 | -0.11, 0.23 | 0.0 | -0.14, 0.18 | 0.3 (1.1) | 0.2 | 0.07, 0.25 | 0.1 | 0.01, 0.18 | |
| Weight/age z-score: | 0.3(1.0) | 0.2(1.1) | -0.1 | -0.36, 0.15 | -0.1 | | -0.38, 0.12 | 0.4(1.3) | 0.0 | -0.14, 0.21 | 0.0 | -0.16, 0.19 | 0.4(1.0) | 0.1 | 0.05, 0.23 | 0.1 | 0.02, 0.19 | |
| Height/age z-score | 0.3 (1.1) | 0.1 (1.0) | -0.2 | -0.44, -0.01 | -0.2 | | -0.46, -0.01 | 0.3 (1.1) | 0.0 | -0.17, 0.17 | 0.0 | -0.18, 0.16 | 0.4 (1.1) | 0.1 | -0.02, 0.15 | 0.1 | -0.03, 0.15 | |
| BMI/age z-score: | 0.2(1.3) | 0.3(1.2) | 0.0 | -0.33, 0.32 | 0.0 | | -0.36, 0.28 | 0.3(1.3) | 0.1 | -0.15, 0.25 | 0.0 | -0.16, 0.22 | 0.4(1.2) | 0.2 | 0.08, 0.28 | 0.1 | 0.03, 0.22 | |
| **BP percentiles (mmHg)** |  |  |  |  |  | |  |  |  |  |  |  |  |  |  |  |  | |
| Systolic | 31.6 (27.6) | 32.5 (27.3) | 1.0 | -5.04, 6.93 | -0.5 | | -6.76, 5.86 | 33.7 (28.3) | 2.1 | -2.29, 6.47 | 1.2 | -3.20, 5.52 | 33.1 (29.2) | 1.5 | -0.88, 3.88 | 0.7 | -1.61, 3.01 | |
| Diastolic | 51.2 (23.4) | 51.5 (21.2) | 0.2 | -4.86, 5.32 | -0.7 | | -5.30, 3.97 | 53.8 (21.4) | 2.6 | -0.80, 5.95 | 1.9 | -1.46, 5.29 | 52.8 (23.2) | 1.6 | -0.31, 3.46 | 1.2 | -0.70, 3.02 | |
| HR (beats/min) | 74.7 (10.6) | 76.4 (11.2) | 1.6 | -1.06, 4.29 | 1.2 | | -1.44, 3.80 | 75.3 (11.3) | 0.6 | -1.35, 2.44 | 0.4 | -1.55, 2.25 | 75.8 (10.9) | 1.0 | 0.15, 1.90 | 1.0 | 0.10, 1.79 | |
| **Physical fitness** |  |  |  |  |  | |  |  |  |  |  |  |  |  |  |  |  | |
| Jump length (m) | 1.2 (0.2) | 1.2 (0.3) | 0.0 | -0.08, 0.05 | 0.0 | | -0.06, 0.07 | 1.2 (0.2) | 0.0 | -0.08, 0.00 | 0.0 | -0.07, 0.01 | 1.2 (0.2) | 0.0 | -0.02, 0.02 | 0.0 | -0.01, 0.03 | |
| N of sit-ups | 11.6 (4.3) | 11.8 (4.7) | 0.2 | -1.00, 1.37 | 0.3 | | -0.96, 1.51 | 11.1 (4.7) | -0.5 | -1.29, 0.29 | -0.5 | -1.23, 0.17 | 11.5 (4.6) | -0.1 | -0.47, 0.28 | 0.0 | -0.34, 0.37 | |
| **Cognitive/intelligence** |  |  |  |  |  | |  |  |  |  |  |  |  |  |  |  |  | |
| Matrix FSIQ score ^a^ | 10.0 (2.5) | 8.9 (2.8) | -1.0 | -1.74, -0.33 | -0.7 | | -1.46, 0.01 | 9.9 (2.4) | -0.1 | -0.47, 0.31 | 0.1 | -0.31, 0.47 | 9.9 (2.5) | -0.1 | -0.31, 0.11 | 0.0 | -0.19, 0.21 | |
| Puzzle PIQ score ^a^ | 10.5 (2.5) | 9.3 (2.6) | -1.3 | -1.95, -0.55 | -0.9 | | -1.53, -0.27 | 10.2 (2.4) | -0.3 | -0.71, 0.08 | -0.1 | -0.48, 0.28 | 10.3 (2.4) | -0.2 | -0.40, -0.01 | -0.1 | -0.28, 0.10 | |
| PPVT score ^b^ | 98.0 (12.8) | 94.0 (13.2) | -3.9 | -6.81, -1.08 | -2.5 | | -5.37, 0.35 | 96.5 (14.2) | -1.5 | -4.00, 0.95 | -0.5 | -2.98, 1.89 | 97.2 (12.8) | -0.8 | -1.82, 0.29 | 0.0 | -0.97, 0.96 | |
|  |  |  |  |  |  | |  |  |  |  |  |  |  |  |  |  |  | |
|  | **%** | **%** | **RR** | **95% CI** | **RR** | **95% CI** | | **%** | **RR** | **95% CI** | **RR** | **95% CI** | **%** | **RR** | **95% CI** | **RR** | | **95% CI** |
| **Motor skills** |  |  |  |  |  |  | |  |  |  |  |  |  |  |  |  | |  |
| Dribbling (0-1/4) | 19.0% | 15.6% | 0.82 | 0.51, 1.31 | 0.72 | 0.42, 1.23 | | 17.9% | 0.95 | 0.68, 1.32 | 0.93 | 0.66, 1.32 | 21.6% | 1.14 | 0.97, 1.34 | 1.12 | | 0.96, 1.31 |
| One leg (0-2/5) | 5.5% | 11.4% | 2.08 | 0.86, 5.02 | 2.05 | 0.92, 4.54 | | 10.5% | 1.90 | 1.14, 3.19 | 1.73 | 1.04, 2.87 | 6.4% | 1.17 | 0.85, 1.60 | 1.14 | | 0.83, 1.56 |
| Throwing a ball (0-1/4) | 23.8% | 27.0% | 1.13 | 0.74, 1.74 | 1.04 | 0.68, 1.60 | | 25.5% | 1.07 | 0.81, 1.42 | 1.03 | 0.77, 1.37 | 25.0% | 1.05 | 0.91, 1.21 | 1.03 | | 0.90, 1.19 |
| Jumping (0-1/4) | 10.8% | 11.0% | 1.01 | 0.55, 1.85 | 0.89 | 0.45, 1.77 | | 12.2% | 1.13 | 0.72, 1.79 | 1.05 | 0.65, 1.69 | 14.8% | 1.37 | 1.10, 1.72 | 1.34 | | 1.08, 1.67 |
| Global score (0-8/17) | 3.5% | 4.8% | 1.38 | 0.62, 3.05 | 1.38 | 0.56, 3.40 | | 5.1% | 1.46 | 0.69, 3.06 | 1.39 | 0.68, 2.88 | 5.2% | 1.48 | 0.99, 2.21 | 1.40 | | 0.94, 2.09 |

Linear regression was performed for continuous outcomes (β, 95% CI) and the quasi Poisson regression for binomial outcomes (RR, 95% CI). Analyses adjusted for: mother’s age, country of birth, education level, employment status; household income, and CSP; mother’s history of diabetes mellitus, arterial hypertension, infertility treatment, pre-pregnancy BMI, and smoking during pregnancy; foetal growth restriction, child’s sex

^a^ Standardised score from the Wechsler Intelligence Scale for Children V (WISC-V) matrix of the full-scale IQ (FSIQ) and puzzle of the performance IQ (PIQ) scale subtests; ^b^ Peabody Picture Vocabulary Test (PPVT) 5, adapted to French, assessing children’s knowledge of spoken words and receptive vocabulary

eTable 3 Descriptive analysis (prevalence) and outcome wide regression results of reported outcomes from the weighted telephone interview ELFE subsample, N = 7824 – adding maternal mental health during pregnancy as a potential confounder

|  | 39 – 40 GA | **32 – 33 GA, N = 41** | | | | | **34 – 36 GA, N = 419** | | | | | **37 - 38 GA, N = 1898** | | | | |
| --- | --- | --- | --- | --- | --- | --- | --- | --- | --- | --- | --- | --- | --- | --- | --- | --- |
|  | N = 5466 | *Main analysis adjusted model* | | | *+ mother’s mental health* | | *Main analysis adjusted model* | | | *+ mother’s mental health* | | *Main analysis adjusted model* | | | *+ mother’s mental health* | |
| OUTCOMES |  | % | RR | 95% CI | RR | 95% CI | % | RR | 95% CI | RR | 95% CI | % | RR | 95% CI | RR | 95% CI |
| **Respiratory & allergies** |  |  |  |  |  |  |  |  |  |  |  |  |  |  |  |  |
| Asthma | 6.2% | 7.3% | 1.24 | 0.35, 4.45 | 1.31 | 0.37, 4.67 | 8.5% | 1.21 | 0.81, 1.83 | 1.23 | 0.81, 1.85 | 7.7% | 1.20 | 0.94, 1.54 | 1.20 | 0.94, 1.53 |
| Eczema | 24.8% | 26.4% | 1.09 | 0.56, 2.15 | 1.10 | 0.57, 2.11 | 26.2% | 1.08 | 0.87, 1.34 | 1.09 | 0.87, 1.36 | 25.1% | 1.03 | 0.92, 1.16 | 1.03 | 0.91, 1.16 |
| Food allergies | 4.3% | 5.6% | 1.15 | 0.23, 5.74 | 1.17 | 0.24, 5.82 | 3.9% | 0.87 | 0.49, 1.52 | 0.87 | 0.50, 1.52 | 4.2% | 0.97 | 0.71, 1.33 | 0.98 | 0.71, 1.34 |
| Allergic rhinitis | 18.9% | 17.8% | 0.94 | 0.40, 2.22 | 0.98 | 0.42, 2.29 | 18.0% | 0.93 | 0.70, 1.23 | 0.92 | 0.69, 1.23 | 20.9% | 1.08 | 0.95, 1.24 | 1.09 | 0.95, 1.25 |
| **Puberty signs** |  |  |  |  |  |  |  |  |  |  |  |  |  |  |  |  |
| Period | 6.5% | 0.3% | 0.00 | 0.00, 0.01 | 0.00 | 0.00, 0.01 | 6.4% | 0.95 | 0.44, 2.03 | 0.94 | 0.43, 2.05 | 7.9% | 1.13 | 0.79, 1.62 | 1.13 | 0.78, 1.62 |
| Breast development | 67.5% | 68.3% | 0.89 | 0.64, 1.24 | 0.93 | 0.71, 1.21 | 61.4% | 0.89 | 0.77, 1.02 | 0.88 | 0.76, 1.02 | 67.4% | 0.98 | 0.92, 1.05 | 0.98 | 0.91, 1.05 |
| Precocious puberty* | 4.9% | 3.7% | 0.50 | 0.09, 2.68 | 0.52 | 0.10, 2.79 | 5.3% | 1.01 | 0.59, 1.74 | 1.01 | 0.59, 1.75 | 6.2% | 1.20 | 0.91, 1.60 | 1.20 | 0.90, 1.59 |
| **Visual and dental** |  |  |  |  |  |  |  |  |  |  |  |  |  |  |  |  |
| Wears eyeglasses | 44.7% | 37.5% | 0.79 | 0.45, 1.39 | 0.83 | 0.48, 1.44 | 54.5% | 1.23 | 1.08, 1.40 | 1.22 | 1.07, 1.40 | 44.1% | 0.98 | 0.91, 1.06 | 0.98 | 0.91, 1.05 |
| Strabismus | 1.3% | 0.1% | 0.00 | 0.00, 0.00 | 0.00 | 0.00, 0.00 | 2.1% | 1.54 | 0.72, 3.31 | 1.50 | 0.69, 3.23 | 3.0% | 2.17 | 1.36, 3.46 | 2.11 | 1.34, 3.34 |
| Astigmatism | 13.9% | 9.3% | 0.62 | 0.20, 1.95 | 0.67 | 0.22, 2.06 | 19.6% | 1.43 | 1.06, 1.93 | 1.45 | 1.08, 1.95 | 13.1% | 0.95 | 0.80, 1.12 | 0.94 | 0.80, 1.11 |
| Hyperopia | 14.6% | 12.0% | 0.77 | 0.31, 1.90 | 0.82 | 0.33, 2.01 | 20.3% | 1.42 | 1.10, 1.83 | 1.42 | 1.10, 1.83 | 15.1% | 1.04 | 0.89, 1.22 | 1.04 | 0.88, 1.21 |
| Myopia | 13.7% | 21.6% | 1.60 | 0.72, 3.56 | 1.67 | 0.76, 3.66 | 13.3% | 0.99 | 0.68, 1.45 | 1.00 | 0.68, 1.46 | 13.2% | 0.96 | 0.80, 1.15 | 0.94 | 0.79, 1.13 |
| Malposition of teeth/jaw | 30.8% | 55.9% | 1.90 | 1.32, 2.74 | 1.91 | 1.32, 2.77 | 27.4% | 0.94 | 0.76, 1.16 | 0.94 | 0.76, 1.16 | 33.5% | 1.12 | 1.01, 1.23 | 1.12 | 1.02, 1.23 |
| **Behaviour and associated complaints** |  |  |  |  |  |  |  |  |  |  |  |  |  |  |  |  |
| **SDQ (Abnormal)** |  |  |  |  |  |  |  |  |  |  |  |  |  |  |  |  |
| Emotional | 21.6% | 18.0% | 0.76 | 0.31, 1.85 | 0.80 | 0.33, 1.95 | 19.1% | 0.87 | 0.66, 1.14 | 0.87 | 0.66, 1.14 | 23.3% | 1.07 | 0.94, 1.22 | 1.06 | 0.94, 1.21 |
| Conduct | 13.3% | 7.5% | 0.45 | 0.10, 2.04 | 0.48 | 0.11, 2.16 | 11.8% | 0.84 | 0.61, 1.17 | 0.84 | 0.61, 1.15 | 14.6% | 1.03 | 0.87, 1.23 | 1.03 | 0.87, 1.23 |
| Hyperactivity | 13.7% | 8.9% | 0.63 | 0.23, 1.77 | 0.66 | 0.24, 1.85 | 13.7% | 0.93 | 0.68, 1.27 | 0.94 | 0.69, 1.28 | 15.8% | 1.09 | 0.92, 1.28 | 1.08 | 0.92, 1.27 |
| Peer relations | 11.9% | 18.9% | 1.39 | 0.57, 3.42 | 1.45 | 0.59, 3.56 | 14.2% | 1.11 | 0.80, 1.54 | 1.11 | 0.80, 1.56 | 11.1% | 0.90 | 0.74, 1.10 | 0.90 | 0.74, 1.10 |
| Global score | 11.0% | 8.7% | 0.65 | 0.17, 2.49 | 0.67 | 0.18, 2.58 | 13.1% | 1.11 | 0.79, 1.55 | 1.10 | 0.78, 1.55 | 13.6% | 1.16 | 0.97, 1.40 | 1.17 | 0.97, 1.40 |
| **Physical complaints** |  |  |  |  |  |  |  |  |  |  |  |  |  |  |  |  |
| Frequent headaches | 50.5% | 58.1% | 1.13 | 0.76, 1.68 | 1.13 | 0.75, 1.69 | 46.1% | 0.92 | 0.80, 1.07 | 0.93 | 0.81, 1.08 | 48.1% | 0.95 | 0.89, 1.02 | 0.95 | 0.89, 1.02 |
| Abdominal pain | 11.5% | 14.7% | 1.31 | 0.48, 3.58 | 1.07 | 0.35, 3.28 | 8.9% | 0.76 | 0.51, 1.13 | 0.76 | 0.51, 1.14 | 11.8% | 1.00 | 0.82, 1.22 | 1.00 | 0.82, 1.22 |
| Constipation | 20.8% | 14.2% | 0.71 | 0.28, 1.80 | 0.74 | 0.30, 1.84 | 22.7% | 1.08 | 0.84, 1.38 | 1.09 | 0.85, 1.39 | 22.3% | 1.07 | 0.94, 1.21 | 1.06 | 0.93, 1.21 |
| **Sleep** |  |  |  |  |  |  |  |  |  |  |  |  |  |  |  |  |
| Duration (< 9h / >12h) | 2.7% | 0.2% | 0.00 | 0.00, 0.01 | 0.00 | 0.00, 0.01 | 1.6% | 0.61 | 0.25, 1.53 | 0.62 | 0.25, 1.54 | 3.0% | 1.09 | 0.72, 1.65 | 1.10 | 0.73, 1.66 |
| Lack (Often/Always) | 13.9% | 10.9% | 0.70 | 0.19, 2.65 | 0.74 | 0.20, 2.74 | 13.6% | 0.97 | 0.70, 1.35 | 0.98 | 0.70, 1.36 | 15.4% | 1.09 | 0.93, 1.29 | 1.10 | 0.93, 1.29 |
| Difficulty falling asleep, (Always/Sometimes) | 22.8% | 12.1% | 0.41 | 0.14, 1.17 | 0.42 | 0.15, 1.20 | 20.5% | 1.03 | 0.84, 1.26 | 1.02 | 0.82, 1.25 | 23.5% | 1.03 | 0.92, 1.14 | 1.02 | 0.92, 1.13 |

The quasi Poisson regression was performed for binomial outcomes. All the binomial outcomes had categories yes/no and reference category was set as “no”, unless stated otherwise.

Analyses adjusted for: mother’s age, country of birth, education level, employment status; household income, and CSP; mother’s history of diabetes mellitus, arterial hypertension, infertility treatment, pre-pregnancy BMI, and smoking during pregnancy; foetal growth restriction, child’s sex

eTable 4 Descriptive analysis (prevalence/mean (SD)) and outcome wide regression results of measured outcomes from the weighted home visit ELFE subsample – adding maternal mental health during pregnancy as a potential confounder

|  | 39 – 40 GA | **32 – 33 GA, N = 26** | | | | | **34 – 36 GA, N = 322** | | | | | **37 - 38 GA, N = 1441** | | | | |
| --- | --- | --- | --- | --- | --- | --- | --- | --- | --- | --- | --- | --- | --- | --- | --- | --- |
|  | N = 4250 | *Main analysis adjusted model* | | | *+ mother’s mental health* | | *Main analysis adjusted model* | | | *+ mother’s mental health* | | *Main analysis adjusted model* | | | *+ mother’s mental health* | |
| OUTCOMES | **Mean (SD)** | **Mean (SD)** | **β** | **95% CI** | **β** | **95% CI** | **Mean (SD)** | **β** | **95% CI** | **β** | **95% CI** | **Mean (SD)** | **β** | **95% CI** | **β** | **95% CI** |
| **Anthropometry** |  |  |  |  |  |  |  |  |  |  |  |  |  |  |  |  |
| Waist/Height | 0.4 (0.1) | 0.4 (0.0) | -0.0 | -0.05, 0.00 | -0.0 | -0.06, 0.00 | 0.5 (0.0) | 0.0 | -0.01, 0.01 | 0.0 | -0.01, 0.01 | 0.5 (0.1) | 0.0 | 0.00, 0.01 | 0.0 | 0.00, 0.01 |
| WH ratio z score | 0.1 (1.1) | -0.4 (0.7) | -0.5 | -1.04, -0.01 | -0.5 | -1.16, 0.09 | 0.2 (1.0) | 0.0 | -0.14, 0.18 | 0.0 | -0.17, 0.17 | 0.2 (1.1) | 0.1 | -0.01, 0.16 | 0.1 | 0.01, 0.18 |
| Weight/age z-score: | 0.3(1.1) | 0.0 (1.0) | -0.3 | -0.77, 0.14 | -0.3 | -0.79, 0.27 | 0.3 (1.1) | -0.1 | -0.22, 0.12 | -0.0 | -0.19, 0.18 | 0.4 (1.1) | 0.1 | 0.01, 0.17 | 0.1 | 0.02, 0.19 |
| Height/age z-score | 0.3 (1.1) | 0.3 (0.8) | -0.1 | -0.43, 0.28 | -0.1 | -0.48, 0.27 | 0.3 (1.0) | -0.0 | -0.20, 0.12 | -0.0 | -0.21, 0.16 | 0.4 (1.1) | 0.1 | -0.02, 0.15 | 0.1 | -0.02, 0.15 |
| BMI/age z-score: | 0.3 (1.2) | -0.3 (1.3) | -0.5 | -1.12, 0.17 | -0.3 | -1.09, 0.40 | 0.2 (1.2) | -0.0 | -0.23, 0.15 | 0.0 | -0.19, 0.21 | 0.4 (1.2) | 0.1 | 0.01, 0.19 | 0.1 | 0.03, 0.22 |
| **BP percentiles (mmHg)** |  |  |  |  |  |  |  |  |  |  |  |  |  |  |  |  |
| Systolic | 31.6 (27.6) | 23.5 (18.8) | -8.7 | -19.15, 1.72 | -8.2 | -19.35, 2.91 | 31.9 (27.6) | -0.7 | -4.73, 3.34 | 1.0 | -3.60, 5.75 | 32.7 (29.0) | 0.4 | -1.87, 2.61 | 0.7 | -1.63, 2.99 |
| Diastolic | 51.3 (23.4) | 48.2 (21.3) | -4.0 | -13.17, 5.16 | -1.5 | -11.77, 8.70 | 51.0 (21.6) | -1.4 | -4.80, 2.04 | 1.3 | -1.77, 5.44 | 52.4 (23.2) | 0.7 | -1.09, 2.56 | 1.2 | -0.64, 3.09 |
| HR (beats/min) | 74.7 (10.6) | 74.9 (10.3) | -0.3 | -4.97, 4.35 | 2.2 | -2.74, 7.18 | 75.7 (10.9) | 0.7 | -1.05, 2.40 | 0.4 | -1.70, 2.39 | 75.7 (10.8) | 1.0 | 0.15, 1.78 | 0.9 | 0.08, 1.77 |
| **Physical fitness** |  |  |  |  |  |  |  |  |  |  |  |  |  |  |  |  |
| Jump length (m) | 1.2 (0.2) | 1.3 (0.3) | 0.1 | -0.07, 0.20 | 0.1 | -0.09, 0.23 | 1.2 (0.2) | -0.0 | -0.08, -0.01 | -0.0 | -0.07, 0.01 | 1.2 (0.2) | 0.0 | -0.01, 0.03 | 0.0 | -0.01, 0.03 |
| N of sit-ups | 11.6 (4.3) | 14.0 (4.1) | 2.3 | 0.10, 4.56 | 3.1 | 0.52, 5.60 | 11.1 (4.7) | -0.4 | -1.12, 0.24 | -0.6 | -1.31, 0.19 | 11.5 (4.6) | 0.0 | -0.33, 0.37 | 0.0 | -0.35, 0.36 |
| **Cognitive functioning** |  |  |  |  |  |  |  |  |  |  |  |  |  |  |  |  |
| Matrix FSIQ score | 10.0 (2.5) | 9.8 (2.4) | -0.2 | -1.57, 1.27 | -0.1 | -1.83, 1.67 | 9.9 (2.3) | 0.1 | -0.28, 0.45 | 0.1 | -0.35, 0.49 | 9.8 (2.6) | -0.0 | -0.24, 0.16 | 0.0 | -0.18, 0.22 |
| Puzzle PIQ score | 10.5 (2.5) | 9.5 (2.7) | -0.9 | -2.10, 0.37 | -1.1 | -2.58, 0.29 | 10.2 (2.3) | -0.0 | -0.37, 0.33 | -0.0 | -0.44, 0.37 | 10.3 (2.4) | -0.1 | -0.32, 0.05 | -0.1 | -0.27, 0.11 |
| PPVT score | 98.0 (12.8) | 101.3 (11.6) | 3.4 | -2.04, 8.80 | 1.8 | -3.93, 7.58 | 96.4 (14.4) | -0.4 | -2.54, 1.85 | -0.3 | -2.92, 2.32 | 97.1 (12.7) | -0.2 | -1.10, 0.76 | 0.0 | -0.94, 0.99 |
|  |  |  |  |  |  |  |  |  |  |  |  |  |  |  |  |  |
|  | **%** | **%** | **RR** | **95% CI** | **RR** | **95% CI** | **%** | **RR** | **95% CI** | **RR** | **95% CI** | **%** | **RR** | **95% CI** | **RR** | **95% CI** |
| **Motor skills** |  |  |  |  |  |  |  |  |  |  |  |  |  |  |  |  |
| Dribbling (0-1/4) | 19.0% | 8.1% | 0.72 | 0.42, 1.23 | 0.36 | 0.08, 1.57 | 22.1% | 0.93 | 0.66, 1.32 | 0.92 | 0.63, 1.34 | 21.7% | 1.12 | 0.96, 1.31 | 1.12 | 0.95, 1.31 |
| One leg (0-2/5) | 5.5% | 15.5% | 2.05 | 0.92, 4.54 | 3.59 | 0.98, 13.08 | 10.8% | 1.73 | 1.04, 2.87 | 1.68 | 0.97, 2.89 | 6.3% | 1.14 | 0.83, 1.56 | 1.13 | 0.83, 1.56 |
| Throwing a ball (0-1/4) | 23.8% | 32.5% | 1.04 | 0.68, 1.60 | 1.30 | 0.60, 2.84 | 28.0% | 1.03 | 0.77, 1.37 | 1.00 | 0.73, 1.37 | 25.1% | 1.03 | 0.90, 1.19 | 1.03 | 0.90, 1.18 |
| Jumping (0-1/4) | 10.8% | 5.7% | 0.89 | 0.45, 1.77 | 0.32 | 0.03, 3.23 | 13.1% | 1.05 | 0.65, 1.69 | 1.02 | 0.60, 1.72 | 14.6% | 1.34 | 1.08, 1.67 | 1.34 | 1.08, 1.67 |
| Global score (0-8/17) | 3.5% | 3.1% | 1.38 | 0.56, 3.40 | 0.99 | 0.10, 9.28 | 5.9% | 1.39 | 0.68, 2.88 | 1.43 | 0.66, 3.09 | 4.9% | 1.40 | 0.94, 2.09 | 1.40 | 0.94, 2.09 |

Analyses adjusted for: mother’s age, country of birth, education level, employment status; household income, and CSP; mother’s history of diabetes mellitus, arterial hypertension, infertility treatment, pre-pregnancy BMI, and smoking during pregnancy; foetal growth restriction, child’s sex
