# Supplementary material for: Health and Development of Children Born Moderate and Late Preterm and Early Term at Age 10 in French Birth Cohorts ELFE and EPIPAGE 2
Source: Paediatr Perinat Epidemiol. 2025 Sep 29;40(1):34–52. doi: 10.1111/ppe.70069 (PMC12853227; doi:10.1111/ppe.70069)
Supplement: Supplementary file 1 — Data S1: ppe70069‐sup‐0001‐Supinfo01.zip. [file PPE-40-34-s001.zip › Appendix 4 Supplemental tables.docx]

Appendix 4 Supplemental tables

eTable 1 Descriptive analysis of participants from ELFE and EPIPAGE 2 cohorts, collected at birth (inclusion), stratified by gestational age and inclusion in the telephone interview subsample

|  | **32-33 GA, MPT** | | | **34-36 GA, LPT** | | | **37-38 GA, ET** | | | **39-40 GA, FT** | | | **Missing for GA** | | |
| --- | --- | --- | --- | --- | --- | --- | --- | --- | --- | --- | --- | --- | --- | --- | --- |
| **Characteristic** | Missing data | Excluded N = 331*^a^* | Included N = 325*^a^* | Missing data | Excluded N = 768*^a^* | Included N = 670*^a^* | Missing data | Excluded N = 1863*^a^* | Included N = 1865*^a^* | Missing data | Excluded N = 4604*^a^* | Included N = 5369*^a^* | Missing data | Excluded N = 248*^a^* | Included N = 144*^a^* |
| **Mother’s age at birth** | 0.0% (0) | 29.4 (5.4) | 31.3 (4.9) | 0.0% (0) | 29.1 (5.7) | 31.2 (5.0) | 0.1% (5) | 29.6 (5.5) | 31.3 (4.6) | 0.1% (11) | 29.0 (5.5) | 30.9 (4.5) | 24.2% (95) | 30.0 (5.0) | 32.2 (4.4) |
| **Mother’s age categorical** |  |  |  |  |  |  |  |  |  |  |  |  |  |  |  |
| ≤ 24 |  | 16.0% (53) | 6.8% (22) |  | 22.5% (173) | 7.5% (50) |  | 19.7% (366) | 6.4% (119) |  | 21.7% (995) | 6.7% (357) |  | 12.9% (20) | 4.2% (6) |
| 25-34 |  | 66.5% (220) | 66.2% (215) |  | 57.7% (443) | 66.1% (443) |  | 60.9% (1132) | 70.0% (1306) |  | 61.3% (2818) | 72.2% (3876) |  | 70.3% (109) | 64.1% (91) |
| ≥ 35 |  | 17.5% (58) | 27.1% (88) |  | 19.8% (152) | 26.4% (177) |  | 19.4% (360) | 23.6% (440) |  | 17.0% (781) | 21.1% (1135) |  | 16.8% (26) | 31.7% (45) |
| **Mother’s country of birth** | 0.8% (5) |  |  | 0.3% (4) |  |  | 0.3% (12) |  |  | 0.3% (29) |  |  | 23.5% (92) |  |  |
| France |  | 75.8% (247) | 88.3% (287) |  | 81.0% (620) | 90.6% (606) |  | 80.5% (1,490) | 91.0% (1698) |  | 82.3% (3,767) | 91.2% (4898) |  | 76.4% (120) | 91.6% (131) |
| Other European countries |  | 4.9% (16) | 0.6% (2) |  | 3.5% (27) | 1.8% (12) |  | 2.3% (43) | 2.0% (38) |  | 2.3% (106) | 2.2% (119) |  | 3.8% (6) | 2.1% (3) |
| North African countries |  | 6.7% (22) | 4.9% (16) |  | 7.6% (58) | 2.1% (14) |  | 8.3% (153) | 2.3% (42) |  | 7.6% (349) | 2.4% (129) |  | 9.6% (15) | 3.5% (5) |
| Other African countries |  | 7.7% (25) | 2.8% (9) |  | 5.1% (39) | 2.8% (19) |  | 6.4% (118) | 2.8% (52) |  | 4.8% (219) | 2.4% (127) |  | 6.4% (10) | 1.4% (2) |
| Other countries |  | 4.9% (16) | 3.4% (11) |  | 2.7% (21) | 2.7% (18) |  | 2.5% (47) | 1.9% (35) |  | 2.9% (134) | 1.8% (96) |  | 3.8% (6) | 1.4% (2) |
| **Mothers’ education level** | 5.8% (38) |  |  | 3.5% (50) |  |  | 0.1% (5) |  |  | 0.1% (13) |  |  | 26.5% (104) |  |  |
| ≤ Primary education |  | 13.6% (41) | 3.5% (11) |  | 14.8% (107) | 4.9% (33) |  | 14.0% (260) | 3.8% (70) |  | 11.9% (547) | 3.2% (170) |  | 11.3% (17) | 3.6% (5) |
| High school |  | 46.8% (141) | 29.7% (94) |  | 48.3% (348) | 33.4% (223) |  | 49.9% (927) | 30.0% (560) |  | 49.6% (2,278) | 26.6% (1429) |  | 54.3% (82) | 14.6% (20) |
| ≤ 2 years of University |  | 15.0% (45) | 26.2% (83) |  | 14.0% (101) | 26.1% (174) |  | 14.0% (260) | 22.4% (417) |  | 13.9% (640) | 22.4% (1205) |  | 10.6% (16) | 20.4% (28) |
| > 2 years of University |  | 24.6% (74) | 40.7% (129) |  | 22.9% (165) | 35.5% (237) |  | 22.1% (411) | 43.9% (818) |  | 24.5% (1,127) | 47.8% (2564) |  | 23.8% (36) | 61.3% (84) |
| **Mother’s employment** | 3.0% (20) |  |  | 3.4% (49) |  |  | 2.7% (99) |  |  | 2.6% (264) |  |  | 37.8% (148) |  |  |
| Professional activity |  | 56.6% (180) | 79.9% (254) |  | 65.9% (486) | 82.1% (535) |  | 65.5% (1,173) | 84.9% (1562) |  | 67.6% (2,976) | 86.7% (4600) |  | 69.7% (85) | 88.5% (108) |
| Unemployed |  | 11.6% (37) | 6.6% (21) |  | 9.2% (68) | 5.1% (33) |  | 8.7% (156) | 4.5% (82) |  | 7.8% (344) | 3.9% (205) |  | 5.7% (7) | 3.3% (4) |
| Stay at home parent |  | 22.3% (71) | 9.4% (30) |  | 16.7% (123) | 7.1% (46) |  | 16.9% (302) | 4.5% (83) |  | 15.3% (673) | 4.4% (235) |  | 18.9% (23) | 4.1% (5) |
| Student |  | 3.1% (10) | 1.6% (5) |  | 2.4% (18) | 2.3% (15) |  | 3.2% (57) | 2.0% (37) |  | 3.8% (166) | 1.8% (97) |  | 1.6% (2) | 0.8% (1) |
| Other |  | 6.3% (20) | 2.5% (8) |  | 5.7% (42) | 3.5% (23) |  | 5.7% (102) | 4.1% (75) |  | 5.6% (245) | 3.2% (168) |  | 4.1% (5) | 3.3% (4) |
| **Household CSP ^b^** | 4.1% (27) |  |  | 3.3% (47) |  |  | 1.4% (52) |  |  | 1.3% (129) |  |  | 26.8% (105) |  |  |
| Executive |  | 17.5% (55) | 29.3% (92) |  | 17.0% (125) | 30.0% (197) |  | 15.6% (282) | 36.3% (676) |  | 18.0% (807) | 38.0% (2039) |  | 20.0% (29) | 43.0% (61) |
| Middleman professions |  | 13.7% (43) | 21.0% (66) |  | 15.4% (113) | 25.4% (167) |  | 18.2% (330) | 24.2% (451) |  | 18.2% (816) | 25.6% (1375) |  | 18.6% (27) | 26.1% (37) |
| Administrative, public service, student |  | 15.9% (50) | 10.2% (32) |  | 11.2% (82) | 9.4% (62) |  | 10.7% (194) | 7.6% (141) |  | 11.7% (526) | 7.2% (387) |  | 6.9% (10) | 4.9% (7) |
| Service job, commerce |  | 38.7% (122) | 32.8% (103) |  | 44.1% (324) | 31.8% (209) |  | 48.2% (874) | 30.1% (561) |  | 45.8% (2,053) | 27.8% (1493) |  | 48.3% (70) | 25.4% (36) |
| Manual worker |  | 12.7% (40) | 6.1% (19) |  | 9.7% (71) | 3.2% (21) |  | 7.0% (127) | 1.6% (30) |  | 5.5% (248) | 1.2% (66) |  | 4.8% (7) | 0.7% (1) |
| Without profession |  | 1.6% (5) | 0.6% (2) |  | 2.6% (19) | 0.2% (1) |  | 0.3% (6) | 0.2% (4) |  | 0.6% (28) | 0.1% (6) |  | 1.4% (2) | 0.0% (0) |
| **Household income ^c^** | 26.5% (174) |  |  | 22.0% (310) |  |  | 15.0% (558) |  |  | 14.0% (1,372) |  |  | 38.8% (150) |  |  |
| < 1500 € |  | 22.3% (50) | 6.6% (17) |  | 19.0% (103) | 5.6% (33) |  | 13.7% (189) | 4.0% (71) |  | 15.8% (541) | 3.8% (198) |  | 15.9% (17) | 2.2% (3) |
| [1500€ - 4000 €] |  | 62.9% (141) | 69.0% (178) |  | 69.1% (375) | 69.2% (405) |  | 68.3% (940) | 64.2% (1152) |  | 66.0% (2,266) | 63.4% (3278) |  | 62.6% (67) | 57.8% (78) |
| > 4000 € |  | 14.7% (33) | 24.4% (63) |  | 12.0% (65) | 25.1% (147) |  | 18.0% (247) | 31.8% (571) |  | 18.2% (626) | 32.7% (1692) |  | 21.5% (23) | 40.0% (54) |
| **Mother’s pre-pregnancy BMI** | 2.6% (17) |  |  | 1.7% (25) |  |  | 1.6% (61) |  |  | 1.6% (155) |  |  | 38.0% (149) |  |  |
| < 18.5 |  | 14.4% (46) | 6.0% (19) |  | 10.4% (78) | 9.8% (65) |  | 9.1% (166) | 7.5% (138) |  | 9.1% (408) | 7.4% (394) |  | 9.7% (12) | 10.1% (12) |
| 18.5 - 24.9 |  | 57.5% (184) | 62.7% (200) |  | 62.3% (467) | 65.2% (433) |  | 58.1% (1,059) | 68.5% (1263) |  | 62.6% (2,822) | 69.1% (3669) |  | 60.5% (75) | 67.2% (80) |
| 25.0 - 29.9 |  | 18.1% (58) | 17.9% (57) |  | 15.4% (115) | 14.0% (93) |  | 19.3% (351) | 15.8% (291) |  | 17.4% (783) | 15.8% (841) |  | 16.9% (21) | 17.6% (21) |
| >=30.0 |  | 10.0% (32) | 13.5% (43) |  | 11.9% (89) | 11.0% (73) |  | 13.5% (247) | 8.2% (152) |  | 10.9% (493) | 7.7% (408) |  | 12.9% (16) | 5.0% (6) |
| **Gestational DM ^d^** | 6.9% (45) | 14.2% (44) | 10.6% (32) | 5.6% (81) | 11.6% (83) | 10.0% (64) | 4.6% (173) | 12.5% (224) | 10.1% (179) | 4.0% (398) | 6.9% (306) | 6.7% (344) | 74.5% (292) | 8.5% (4) | 3.8% (2) |
| **History of DM** | 1.1% (7) | 2.5% (8) | 0.3% (1) | 1.9% (27) | 1.9% (14) | 2.3% (15) | 2.0% (74) | 2.0% (36) | 1.7% (31) | 2.0% (199) | 0.5% (24) | 0.9% (47) | 72.4% (284) | 0.0% (0) | 1.8% (1) |
| **Gestational HTA ^e^** | 1.4% (9) | 20.6% (67) | 22.7% (73) | 2.1% (30) | 13.4% (101) | 12.9% (85) | 2.0% (73) | 5.0% (92) | 5.0% (91) | 1.7% (167) | 2.6% (119) | 2.6% (136) | 75.0% (294) | 2.1% (1) | 3.9% (2) |
| **History of HTA** | 0.6% (4) | 6.4% (21) | 4.9% (16) | 0.8% (11) | 5.1% (39) | 6.4% (43) | 1.4% (51) | 2.5% (45) | 3.1% (58) | 1.2% (115) | 2.1% (94) | 2.0% (108) | 72.2% (283) | 3.8% (2) | 0.0% (0) |
| **Infertility treatment** | 2.3% (15) | 17.0% (55) | 24.2% (77) | 1.8% (26) | 12.0% (90) | 19.7% (130) | 1.6% (59) | 7.5% (138) | 11.3% (208) | 1.5% (146) | 5.3% (241) | 8.8% (468) | 37.2% (146) | 7.3% (9) | 17.2% (21) |
| **Tobacco smoking ^f^** | 2.4% (16) | 24.2% (79) | 12.4% (39) | 2.2% (32) | 31.1% (233) | 16.4% (108) | 1.0% (36) | 27.6% (508) | 17.0% (314) | 1.1% (112) | 24.8% (1124) | 15.1% (803) | 36.2% (142) | 15.0% (19) | 17.1% (21) |
| **Foetal growth restriction ^g^** | 3.2% (21) | 19.9% (64) | 20.4% (64) | 3.8% (54) | 13.4% (98) | 12.3% (80) | 3.5% (132) | 5.8% (105) | 5.0% (89) | 3.4% (337) | 2.8% (125) | 1.9% (100) | 75.0% (294) | 8.3% (4) | 2.0% (1) |
| **Labour type** | 0.5% (3) |  |  | 1.3% (18) |  |  | 1.7% (63) |  |  | 1.3% (131) |  |  | 76.3% (299) |  |  |
| Vaginal delivery |  | 39.0% (128) | 29.5% (96) |  | 50.4% (381) | 53.5% (355) |  | 63.9% (1169) | 64.6% (1186) |  | 71.9% (3,265) | 73.1% (3879) |  | 55.6% (25) | 75.0% (36) |
| Vaginal instrumental |  | 6.4% (21) | 5.8% (19) |  | 7.8% (59) | 7.2% (48) |  | 9.8% (179) | 10.2% (188) |  | 13.3% (602) | 12.4% (660) |  | 15.6% (7) | 4.2% (2) |
| Elective caesarean |  | 39.9% (131) | 44.0% (143) |  | 27.6% (209) | 26.8% (178) |  | 17.3% (317) | 17.5% (322) |  | 6.6% (298) | 7.1% (377) |  | 20.0% (9) | 10.4% (5) |
| Non-elective caesarean |  | 14.6% (48) | 20.6% (67) |  | 14.2% (107) | 12.5% (83) |  | 9.0% (164) | 7.6% (140) |  | 8.2% (373) | 7.3% (388) |  | 8.9% (4) | 10.4% (5) |
| **Multiple births** (Twins) | 0.0% (0) | 38.1% (126) | 38.8% (126) | 0.0% (0) | 25.8% (198) | 27.5% (184) | 0.0% (0) | 8.3% (154) | 7.6% (142) | 0.0% (0) | 0.2% (10) | 0.4% (22) | 15.1% (59) | 5.3% (10) | 5.6% (8) |
| **Resuscitation at birth** (Yes) | 1.8% (12) | 39.4% (128) | 41.4% (132) | 1.3% (18) | 15.4% (117) | 16.8% (111) | 0.0% (0) | 3.2% (60) | 3.1% (58) | 0.0% (0) | 1.9% (86) | 2.3% (126) | 63.8% (250) | 0.0% (0) | 1.4% (1) |
| **Child’s sex** (Female) | 0.0% (0) | 48.3% (160) | 44.0% (143) | 0.0% (0) | 47.1% (362) | 47.3% (317) | 0.0% (0) | 47.2% (880) | 47.0% (877) | 0.0% (1) | 49.0% (2257) | 49.6% (2665) | 25.0% (98) | 44.4% (67) | 51.0% (73) |
| **Birth weight z-score ^h^** | 0.2% (1) |  |  | 0.9% (13) |  |  | 1.3% (50) |  |  | 1.3% (129) |  |  | 100.0% (392) |  |  |
| < -2 |  | 20.0% (66) | 19.7% (64) |  | 11.5% (88) | 10.0% (66) |  | 4.1% (76) | 3.1% (57) |  | 2.4% (107) | 1.5% (82) |  | (0) | (0) |
| [-2, -1> |  | 22.7% (75) | 23.1% (75) |  | 18.2% (139) | 20.2% (134) |  | 15.8% (290) | 13.4% (246) |  | 13.3% (603) | 12.4% (657) |  | (0) | (0) |
| [-1, 0> |  | 33.0% (109) | 28.3% (92) |  | 32.1% (245) | 32.2% (213) |  | 31.8% (584) | 33.4% (616) |  | 33.2% (1,509) | 34.5% (1831) |  | (0) | (0) |
| [0, 1> |  | 16.7% (55) | 21.2% (69) |  | 26.0% (198) | 23.9% (158) |  | 29.1% (535) | 31.3% (576) |  | 33.4% (1,517) | 34.7% (1839) |  | (0) | (0) |
| [1, 2> |  | 4.2% (14) | 6.8% (22) |  | 8.4% (64) | 9.8% (65) |  | 13.6% (249) | 13.6% (250) |  | 13.9% (632) | 13.1% (695) |  | (0) | (0) |
| >= 2 |  | 3.3% (11) | 0.9% (3) |  | 3.8% (29) | 3.9% (26) |  | 5.6% (102) | 5.3% (97) |  | 3.8% (173) | 3.8% (199) |  | (0) | (0) |
| **Exclusive breastfeeding** | 6.6% (43) | 41.4% (126) | 36.2% (112) | 3.5% (50) | 42.1% (310) | 37.8% (246) | 0.4% (16) | 47.5% (881) | 63.2% (1173) | 0.4% (39) | 52.4% (2398) | 70.9% (3798) | 31.4% (123) | 52.9% (73) | 61.1% (80) |
| **Breastfeeding duration ^i^** | 2.0% (13) | 1.2 (2.6) | 2.3 (3.6) | 10.0% (144) | 1.7 (4.0) | 2.5 (4.8) | 15.5% (578) | 2.0 (4.2) | 3.7 (5.3) | 15.8% (1,580) | 2.1 (4.3) | 4.3 (5.9) | 38.0% (149) | 2.1 (3.3) | 4.0 (5.3) |

*^a^* % (n) / mean (SD)

^b^ CP: from French catégorie socioprofessionnelle, meaning socio-professional domain; ^c^ Household income per month; ^d^ DM: Diabetes mellitus; ^e^ HTA: arterial hypertension; ^f^ Tobacco smoking during the second half of pregnancy; ^g^ Intrauterine growth restriction suspected during pregnancy; *^h^* Gardosi birth weight z-scores; ^i^ Breast feeding duration in months, variable collected at multiple follow-ups between 0 and 2 years and harmonized between cohorts

eTable 2 Descriptive analysis (prevalence) and outcome-wide regression results of reported outcomes from the weighted telephone interview subsample of singletons, N = 7891

| OUTCOMES | GESTATIONAL AGE | | | | | | | | | | | | | | | |
| --- | --- | --- | --- | --- | --- | --- | --- | --- | --- | --- | --- | --- | --- | --- | --- | --- |
|  | 39 – 40 GA | 32 – 33 GA, N = 202 | | | | | 34 – 36 GA, N = 496 | | | | | 37 – 38 GA, N = 1754 | | | | |
|  | N = 5439 |  | **Unadjusted** | | **Adjusted** | |  | **Unadjusted** | | **Adjusted** | |  | **Unadjusted** | | **Adjusted** | |
|  | % | **%** | **RR** | **95% CI** | **RR** | **95% CI** | **%** | **RR** | **95% CI** | **RR** | **95% CI** | **%** | **RR** | **95% CI** | **RR** | **95% CI** |
| **Respiratory and allergies** | (1.00) |  |  |  |  |  |  |  |  |  |  |  |  |  |  |  |
| Asthma | 6.2% | 8.2% | 1.31 | 0.70, 2.43 | 1.18 | 0.60, 2.32 | 8.4% | 1.34 | 0.82, 2.19 | 1.25 | 0.80, 1.95 | 7.6% | 1.21 | 0.94, 1.57 | 1.20 | 0.93, 1.55 |
| Eczema | 24.8% | 22.7% | 0.91 | 0.62, 1.35 | 0.97 | 0.65, 1.44 | 26.2% | 1.06 | 0.84, 1.33 | 1.08 | 0.86, 1.36 | 25.4% | 1.03 | 0.91, 1.15 | 1.05 | 0.93, 1.18 |
| Food allergies | 4.3% | 5.8% | 1.33 | 0.61, 2.88 | 1.24 | 0.53, 2.92 | 3.8% | 0.89 | 0.49, 1.62 | 0.86 | 0.46, 1.59 | 4.2% | 0.97 | 0.70, 1.35 | 0.98 | 0.71, 1.36 |
| Allergic rhinitis | 19.0% | 25.0% | 1.32 | 0.96, 1.82 | 1.19 | 0.83, 1.71 | 20.4% | 1.07 | 0.81, 1.43 | 1.04 | 0.78, 1.37 | 21.1% | 1.11 | 0.97, 1.28 | 1.10 | 0.96, 1.26 |
| **Puberty signs** |  |  |  |  |  |  |  |  |  |  |  |  |  |  |  |  |
| Period | 6.6% | 4.7% | 0.71 | 0.22, 2.33 | 0.45 | 0.12, 1.73 | 8.9% | 1.35 | 0.70, 2.64 | 1.29 | 0.64, 2.60 | 7.7% | 1.18 | 0.80, 1.75 | 1.12 | 0.77, 1.62 |
| Breast development | 67.8% | 73.3% | 1.08 | 0.88, 1.32 | 0.96 | 0.79, 1.17 | 63.5% | 0.94 | 0.80, 1.10 | 0.91 | 0.77, 1.06 | 68.2% | 1.01 | 0.94, 1.08 | 0.99 | 0.93, 1.06 |
| Precocious puberty ^a^ | 4.9% | 5.9% | 1.20 | 0.61, 2.38 | 0.92 | 0.43, 1.96 | 6.4% | 1.31 | 0.79, 2.17 | 1.28 | 0.77, 2.12 | 6.3% | 1.29 | 0.96, 1.73 | 1.23 | 0.93, 1.64 |
| **Vision and dental** |  |  |  |  |  |  |  |  |  |  |  |  |  |  |  |  |
| Wears eyeglasses | 44.7% | 44.5% | 0.99 | 0.78, 1.27 | 0.96 | 0.74, 1.24 | 53.3% | 1.19 | 1.04, 1.36 | 1.20 | 1.04, 1.37 | 44.1% | 0.99 | 0.91, 1.07 | 0.98 | 0.91, 1.06 |
| Strabismus | 1.3% | 3.3% | 2.56 | 1.13, 5.80 | 2.22 | 0.91, 5.43 | 2.0% | 1.51 | 0.69, 3.30 | 1.49 | 0.66, 3.39 | 3.0% | 2.31 | 1.42, 3.74 | 2.21 | 1.37, 3.57 |
| Astigmatism | 13.9% | 12.5% | 0.89 | 0.55, 1.44 | 0.92 | 0.57, 1.49 | 17.3% | 1.24 | 0.94, 1.64 | 1.27 | 0.96, 1.68 | 13.2% | 0.95 | 0.80, 1.12 | 0.95 | 0.81, 1.13 |
| Hyperopia | 14.6% | 14.0% | 0.96 | 0.61, 1.49 | 1.02 | 0.65, 1.59 | 21.2% | 1.45 | 1.11, 1.89 | 1.53 | 1.17, 1.99 | 14.9% | 1.02 | 0.87, 1.20 | 1.03 | 0.88, 1.22 |
| Myopia | 13.7% | 18.0% | 1.31 | 0.81, 2.10 | 1.25 | 0.79, 1.97 | 13.7% | 0.99 | 0.69, 1.42 | 0.96 | 0.67, 1.38 | 13.1% | 0.95 | 0.80, 1.14 | 0.93 | 0.78, 1.12 |
| Malposition of teeth/jaw | 30.8% | 40.4% | 1.31 | 1.00, 1.72 | 1.41 | 1.08, 1.85 | 27.9% | 0.90 | 0.73, 1.11 | 0.95 | 0.78, 1.17 | 33.1% | 1.07 | 0.97, 1.18 | 1.10 | 1.00, 1.22 |
| **Behaviour and associated complaints** |  |  |  |  |  |  |  |  |  |  |  |  |  |  |  |  |
| **SDQ score (At risk)** |  |  |  |  |  |  |  |  |  |  |  |  |  |  |  |  |
| Emotional | 21.6% | 24.7% | 1.14 | 0.79, 1.63 | 1.07 | 0.74, 1.54 | 19.3% | 0.89 | 0.68, 1.17 | 0.88 | 0.67, 1.15 | 24.2% | 1.12 | 0.99, 1.27 | 1.10 | 0.97, 1.25 |
| Conduct | 13.2% | 14.9% | 1.11 | 0.67, 1.84 | 0.92 | 0.56, 1.52 | 11.3% | 0.85 | 0.61, 1.19 | 0.80 | 0.57, 1.12 | 15.0% | 1.14 | 0.95, 1.36 | 1.07 | 0.89, 1.28 |
| Hyperactivity | 13.7% | 22.2% | 1.62 | 1.11, 2.38 | 1.51 | 1.03, 2.23 | 13.9% | 1.02 | 0.74, 1.41 | 0.95 | 0.69, 1.32 | 16.0% | 1.17 | 0.99, 1.38 | 1.11 | 0.94, 1.30 |
| Peer relations | 11.8% | 19.9% | 1.67 | 1.03, 2.73 | 1.46 | 0.93, 2.32 | 14.4% | 1.22 | 0.85, 1.74 | 1.11 | 0.79, 1.57 | 11.7% | 0.99 | 0.81, 1.21 | 0.95 | 0.78, 1.15 |
| Global score | 10.9% | 14.9% | 1.35 | 0.82, 2.21 | 1.16 | 0.70, 1.91 | 14.1% | 1.29 | 0.90, 1.83 | 1.20 | 0.86, 1.69 | 14.1% | 1.28 | 1.06, 1.55 | 1.21 | 1.00, 1.46 |
| **Physical complaints** |  |  |  |  |  |  |  |  |  |  |  |  |  |  |  |  |
| Abdominal pain | 50.4% | 48.2% | 0.95 | 0.76, 1.21 | 0.94 | 0.74, 1.19 | 49.3% | 0.98 | 0.85, 1.13 | 0.98 | 0.85, 1.13 | 49.2% | 0.97 | 0.91, 1.05 | 0.97 | 0.90, 1.04 |
| Constipation ^b^ | 11.5% | 17.2% | 1.48 | 0.96, 2.29 | 1.39 | 0.84, 2.31 | 8.8% | 0.77 | 0.51, 1.14 | 0.76 | 0.50, 1.15 | 12.0% | 1.04 | 0.85, 1.26 | 1.00 | 0.82, 1.22 |
| Headaches | 20.9% | 15.6% | 0.75 | 0.49, 1.14 | 0.74 | 0.48, 1.15 | 22.6% | 1.08 | 0.83, 1.41 | 1.07 | 0.82, 1.40 | 22.7% | 1.09 | 0.95, 1.24 | 1.09 | 0.95, 1.24 |
| **Sleep** |  |  |  |  |  |  |  |  |  |  |  |  |  |  |  |  |
| Duration (< 9h / >12h) | 2.7% | 1.0% | 0.36 | 0.10, 1.24 | 0.34 | 0.09, 1.31 | 2.0% | 0.74 | 0.31, 1.74 | 0.69 | 0.28, 1.70 | 3.2% | 1.17 | 0.77, 1.77 | 1.14 | 0.75, 1.73 |
| Lack (Often/Always) | 13.9% | 10.9% | 0.95 | 0.54, 1.66 | 0.93 | 0.52, 1.68 | 13.6% | 0.93 | 0.65, 1.34 | 0.93 | 0.65, 1.32 | 15.4% | 1.12 | 0.95, 1.33 | 1.11 | 0.94, 1.32 |
| Difficulty falling asleep (Always/Sometimes) | 22.8% | 18.1% | 0.77 | 0.51, 1.15 | 0.76 | 0.50, 1.14 | 20.2% | 0.96 | 0.76, 1.22 | 0.94 | 0.75, 1.18 | 23.7% | 1.03 | 0.92, 1.15 | 1.03 | 0.92, 1.15 |

The quasi Poisson regression was performed for binomial outcomes. All the binomial outcomes had categories yes/no and reference category was set as “no”, unless stated otherwise.

Analyses adjusted for: mother’s age, country of birth, education level, employment status; household income, CSP; mother’s history of diabetes mellitus, arterial hypertension, infertility treatment, pre-pregnancy BMI, smoking during pregnancy; foetal growth restriction, child’s sex;

^a^ Precocious puberty is defined as an appearance of pubic hair before the age of 9.5 for boys and 8 years for girls, or breasts before the age of 8, or period before the age of 11. ^b^ Defined as ≤ 2 stools per week;

eTable 3 Descriptive analysis (prevalence/mean (SD)) and outcome-wide regression results of measured outcomes from the weighted home visit subsample of singletons, N = 6064

| OUTCOMES | GESTATIONAL AGE | | | | | | | | | | | | | | | | | |
| --- | --- | --- | --- | --- | --- | --- | --- | --- | --- | --- | --- | --- | --- | --- | --- | --- | --- | --- |
|  | 39 – 40 GA | 32 – 33 GA, N = 136 | | | | | | 34 – 36 GA, N = 369 | | | | | 37 – 38 GA, N = 1334 | | | | | |
|  | N = 4225 |  | **Unadjusted** | | **Adjusted** | | |  | **Unadjusted** | | **Adjusted** | |  | **Unadjusted** | | **Adjusted** | | |
|  | **Mean (SD)** | **Mean (SD)** | **β** | **95% CI** | **β** | | **95% CI** | **Mean (SD)** | **β** | **95% CI** | **β** | **95% CI** | **Mean (SD)** | **β** | **95% CI** | **β** | **95% CI** | |
| **Anthropometry** | (1.00) |  |  |  |  | |  |  |  |  |  |  |  |  |  |  |  | |
| Waist/Height | 0.4 (0.1) | 0.4 (0.1) | 0.00 | -0.01, 0.02 | 0.00 | | -0.01, 0.01 | 0.5 (0.1) | 0.00 | -0.01, 0.01 | 0.00 | -0.01, 0.01 | 0.5 (0.1) | 0.01 | 0.00, 0.01 | 0.00 | 0.00, 0.01 | |
| WH ratio z score | 0.1 (1.1) | 0.1 (1.2) | 0.04 | -0.24, 0.32 | 0.00 | | -0.31, 0.31 | 0.2 (1.1) | 0.06 | -0.11, 0.23 | 0.02 | -0.14, 0.18 | 0.3 (1.1) | 0.16 | 0.07, 0.25 | 0.10 | 0.01, 0.18 | |
| Weight/age z-score: | 0.3(1.0) | 0.2(1.1) | -0.11 | -0.36, 0.15 | -0.13 | | -0.38, 0.12 | 0.4(1.3) | 0.03 | -0.14, 0.21 | 0.02 | -0.16, 0.19 | 0.4(1.0) | 0.14 | 0.05, 0.23 | 0.10 | 0.02, 0.19 | |
| Height/age z-score | 0.3 (1.1) | 0.1 (1.0) | -0.22 | -0.44, -0.01 | -0.24 | | -0.46, -0.01 | 0.3 (1.1) | 0.00 | -0.17, 0.17 | -0.01 | -0.18, 0.16 | 0.4 (1.1) | 0.06 | -0.02, 0.15 | 0.06 | -0.03, 0.15 | |
| BMI/age z-score: | 0.2(1.3) | 0.3(1.2) | -0.01 | -0.33, 0.32 | -0.04 | | -0.36, 0.28 | 0.3(1.3) | 0.05 | -0.15, 0.25 | 0.03 | -0.16, 0.22 | 0.4(1.2) | 0.18 | 0.08, 0.28 | 0.12 | 0.03, 0.22 | |
| **BP percentiles** (mmHg) |  |  |  |  |  | |  |  |  |  |  |  |  |  |  |  |  | |
| Systolic | 31.6 (27.6) | 32.5 (27.3) | 0.95 | -5.04, 6.93 | -0.45 | | -6.76, 5.86 | 33.7 (28.3) | 2.09 | -2.29, 6.47 | 1.16 | -3.20, 5.52 | 33.1 (29.2) | 1.50 | -0.88, 3.88 | 0.70 | -1.61, 3.01 | |
| Diastolic | 51.2 (23.4) | 51.5 (21.2) | 0.23 | -4.86, 5.32 | -0.66 | | -5.30, 3.97 | 53.8 (21.4) | 2.57 | -0.80, 5.95 | 1.91 | -1.46, 5.29 | 52.8 (23.2) | 1.58 | -0.31, 3.46 | 1.16 | -0.70, 3.02 | |
| HR (beats/min) | 74.7 (10.6) | 76.4 (11.2) | 1.61 | -1.06, 4.29 | 1.18 | | -1.44, 3.80 | 75.3 (11.3) | 0.55 | -1.35, 2.44 | 0.35 | -1.55, 2.25 | 75.8 (10.9) | 1.02 | 0.15, 1.90 | 0.95 | 0.10, 1.79 | |
| **Physical fitness** |  |  |  |  |  | |  |  |  |  |  |  |  |  |  |  |  | |
| Jump length (m) | 1.2 (0.2) | 1.2 (0.3) | -0.02 | -0.08, 0.05 | 0.01 | | -0.06, 0.07 | 1.2 (0.2) | -0.04 | -0.08, 0.00 | -0.03 | -0.07, 0.01 | 1.2 (0.2) | 0.00 | -0.02, 0.02 | 0.01 | -0.01, 0.03 | |
| N of sit-ups | 11.6 (4.3) | 11.8 (4.7) | 0.19 | -1.00, 1.37 | 0.27 | | -0.96, 1.51 | 11.1 (4.7) | -0.50 | -1.29, 0.29 | -0.53 | -1.23, 0.17 | 11.5 (4.6) | -0.09 | -0.47, 0.28 | 0.01 | -0.34, 0.37 | |
| **Cognitive/intelligence** |  |  |  |  |  | |  |  |  |  |  |  |  |  |  |  |  | |
| Matrix FSIQ score ^a^ | 10.0 (2.5) | 8.9 (2.8) | -1.04 | -1.74, -0.33 | -0.73 | | -1.46, 0.01 | 9.9 (2.4) | -0.08 | -0.47, 0.31 | 0.08 | -0.31, 0.47 | 9.9 (2.5) | -0.10 | -0.31, 0.11 | 0.01 | -0.19, 0.21 | |
| Puzzle PIQ score ^a^ | 10.5 (2.5) | 9.3 (2.6) | -1.25 | -1.95, -0.55 | -0.90 | | -1.53, -0.27 | 10.2 (2.4) | -0.32 | -0.71, 0.08 | -0.10 | -0.48, 0.28 | 10.3 (2.4) | -0.20 | -0.40, -0.01 | -0.09 | -0.28, 0.10 | |
| PPVT score ^b^ | 98.0 (12.8) | 94.0 (13.2) | -3.94 | -6.81, -1.08 | -2.51 | | -5.37, 0.35 | 96.5 (14.2) | -1.52 | -4.00, 0.95 | -0.54 | -2.98, 1.89 | 97.2 (12.8) | -0.77 | -1.82, 0.29 | -0.01 | -0.97, 0.96 | |
|  |  |  |  |  |  | |  |  |  |  |  |  |  |  |  |  |  | |
|  | **%** | **%** | **RR** | **95% CI** | **RR** | **95% CI** | | **%** | **RR** | **95% CI** | **RR** | **95% CI** | **%** | **RR** | **95% CI** | **RR** | | **95% CI** |
| **Motor skills** |  |  |  |  |  |  | |  |  |  |  |  |  |  |  |  | |  |
| Dribbling (0-1/4) | 19.0% | 15.6% | 0.82 | 0.51, 1.31 | 0.72 | 0.42, 1.23 | | 17.9% | 0.95 | 0.68, 1.32 | 0.93 | 0.66, 1.32 | 21.6% | 1.14 | 0.97, 1.34 | 1.12 | | 0.96, 1.31 |
| One leg (0-2/5) | 5.5% | 11.4% | 2.08 | 0.86, 5.02 | 2.05 | 0.92, 4.54 | | 10.5% | 1.90 | 1.14, 3.19 | 1.73 | 1.04, 2.87 | 6.4% | 1.17 | 0.85, 1.60 | 1.14 | | 0.83, 1.56 |
| Throwing a ball (0-1/4) | 23.8% | 27.0% | 1.13 | 0.74, 1.74 | 1.04 | 0.68, 1.60 | | 25.5% | 1.07 | 0.81, 1.42 | 1.03 | 0.77, 1.37 | 25.0% | 1.05 | 0.91, 1.21 | 1.03 | | 0.90, 1.19 |
| Jumping (0-1/4) | 10.8% | 11.0% | 1.01 | 0.55, 1.85 | 0.89 | 0.45, 1.77 | | 12.2% | 1.13 | 0.72, 1.79 | 1.05 | 0.65, 1.69 | 14.8% | 1.37 | 1.10, 1.72 | 1.34 | | 1.08, 1.67 |
| Global score (0-8/17) | 3.5% | 4.8% | 1.38 | 0.62, 3.05 | 1.38 | 0.56, 3.40 | | 5.1% | 1.46 | 0.69, 3.06 | 1.39 | 0.68, 2.88 | 5.2% | 1.48 | 0.99, 2.21 | 1.40 | | 0.94, 2.09 |

Linear regression was performed for continuous outcomes (β, 95% CI) and the quasi Poisson regression for binomial outcomes (RR, 95% CI). Analyses adjusted for: mother’s age, country of birth, education level, employment status; household income, CSP; mother’s history of diabetes mellitus, arterial hypertension, infertility treatment, pre-pregnancy BMI, smoking during pregnancy; foetal growth restriction, child’s sex;

^a^ Standard score (mean = 10, SD = 3) from the matrix subtest of Fluid Reasoning Index (FRI) and puzzle subtest of the Visual Spatial Index (VSI) of the WISC-V; ^b^ Peabody Picture Vocabulary Test (PPVT) 5, adapted to French, assessing children’s knowledge of spoken words and receptive vocabulary;

eTable 4 Descriptive analysis (prevalence) and outcome wide regression results of reported outcomes from the weighted telephone interview additional adjustment subsample,

N = 7824

|  | **39–40 GA** | **32–33 GA, N = 41** | | | | | **34–36 GA, N = 419** | | | | | **37-38 GA, N = 1898** | | | | |
| --- | --- | --- | --- | --- | --- | --- | --- | --- | --- | --- | --- | --- | --- | --- | --- | --- |
|  | N = 5466 | *Main analysis adjusted model* | | | *+ mother’s mental health* | | *Main analysis adjusted model* | | | *+ mother’s mental health* | | *Main analysis adjusted model* | | | *+ mother’s mental health* | |
| OUTCOMES |  | % | RR | 95% CI | RR | 95% CI | % | RR | 95% CI | RR | 95% CI | % | RR | 95% CI | RR | 95% CI |
| **Respiratory & allergies** |  |  |  |  |  |  |  |  |  |  |  |  |  |  |  |  |
| Asthma | 6.2% | 7.3% | 1.24 | 0.35, 4.45 | 1.31 | 0.37, 4.67 | 8.5% | 1.21 | 0.81, 1.83 | 1.23 | 0.81, 1.85 | 7.7% | 1.20 | 0.94, 1.54 | 1.20 | 0.94, 1.53 |
| Eczema | 24.8% | 26.4% | 1.09 | 0.56, 2.15 | 1.10 | 0.57, 2.11 | 26.2% | 1.08 | 0.87, 1.34 | 1.09 | 0.87, 1.36 | 25.1% | 1.03 | 0.92, 1.16 | 1.03 | 0.91, 1.16 |
| Food allergies | 4.3% | 5.6% | 1.15 | 0.23, 5.74 | 1.17 | 0.24, 5.82 | 3.9% | 0.87 | 0.49, 1.52 | 0.87 | 0.50, 1.52 | 4.2% | 0.97 | 0.71, 1.33 | 0.98 | 0.71, 1.34 |
| Allergic rhinitis | 18.9% | 17.8% | 0.94 | 0.40, 2.22 | 0.98 | 0.42, 2.29 | 18.0% | 0.93 | 0.70, 1.23 | 0.92 | 0.69, 1.23 | 20.9% | 1.08 | 0.95, 1.24 | 1.09 | 0.95, 1.25 |
| **Puberty signs** |  |  |  |  |  |  |  |  |  |  |  |  |  |  |  |  |
| Period | 6.5% | 0.3% | 0.00 | 0.00, 0.01 | 0.00 | 0.00, 0.01 | 6.4% | 0.95 | 0.44, 2.03 | 0.94 | 0.43, 2.05 | 7.9% | 1.13 | 0.79, 1.62 | 1.13 | 0.78, 1.62 |
| Breast development | 67.5% | 68.3% | 0.89 | 0.64, 1.24 | 0.93 | 0.71, 1.21 | 61.4% | 0.89 | 0.77, 1.02 | 0.88 | 0.76, 1.02 | 67.4% | 0.98 | 0.92, 1.05 | 0.98 | 0.91, 1.05 |
| Precocious puberty ^a^ | 4.9% | 3.7% | 0.50 | 0.09, 2.68 | 0.52 | 0.10, 2.79 | 5.3% | 1.01 | 0.59, 1.74 | 1.01 | 0.59, 1.75 | 6.2% | 1.20 | 0.91, 1.60 | 1.20 | 0.90, 1.59 |
| **Visual and dental** |  |  |  |  |  |  |  |  |  |  |  |  |  |  |  |  |
| Wears eyeglasses | 44.7% | 37.5% | 0.79 | 0.45, 1.39 | 0.83 | 0.48, 1.44 | 54.5% | 1.23 | 1.08, 1.40 | 1.22 | 1.07, 1.40 | 44.1% | 0.98 | 0.91, 1.06 | 0.98 | 0.91, 1.05 |
| Strabismus | 1.3% | 0.1% | 0.00 | 0.00, 0.00 | 0.00 | 0.00, 0.00 | 2.1% | 1.54 | 0.72, 3.31 | 1.50 | 0.69, 3.23 | 3.0% | 2.17 | 1.36, 3.46 | 2.11 | 1.34, 3.34 |
| Astigmatism | 13.9% | 9.3% | 0.62 | 0.20, 1.95 | 0.67 | 0.22, 2.06 | 19.6% | 1.43 | 1.06, 1.93 | 1.45 | 1.08, 1.95 | 13.1% | 0.95 | 0.80, 1.12 | 0.94 | 0.80, 1.11 |
| Hyperopia | 14.6% | 12.0% | 0.77 | 0.31, 1.90 | 0.82 | 0.33, 2.01 | 20.3% | 1.42 | 1.10, 1.83 | 1.42 | 1.10, 1.83 | 15.1% | 1.04 | 0.89, 1.22 | 1.04 | 0.88, 1.21 |
| Myopia | 13.7% | 21.6% | 1.60 | 0.72, 3.56 | 1.67 | 0.76, 3.66 | 13.3% | 0.99 | 0.68, 1.45 | 1.00 | 0.68, 1.46 | 13.2% | 0.96 | 0.80, 1.15 | 0.94 | 0.79, 1.13 |
| Malposition of teeth/jaw | 30.8% | 55.9% | 1.90 | 1.32, 2.74 | 1.91 | 1.32, 2.77 | 27.4% | 0.94 | 0.76, 1.16 | 0.94 | 0.76, 1.16 | 33.5% | 1.12 | 1.01, 1.23 | 1.12 | 1.02, 1.23 |
| **Behaviour and associated complaints** |  |  |  |  |  |  |  |  |  |  |  |  |  |  |  |  |
| **SDQ** (At risk) |  |  |  |  |  |  |  |  |  |  |  |  |  |  |  |  |
| Emotional | 21.6% | 18.0% | 0.76 | 0.31, 1.85 | 0.80 | 0.33, 1.95 | 19.1% | 0.87 | 0.66, 1.14 | 0.87 | 0.66, 1.14 | 23.3% | 1.07 | 0.94, 1.22 | 1.06 | 0.94, 1.21 |
| Conduct | 13.3% | 7.5% | 0.45 | 0.10, 2.04 | 0.48 | 0.11, 2.16 | 11.8% | 0.84 | 0.61, 1.17 | 0.84 | 0.61, 1.15 | 14.6% | 1.03 | 0.87, 1.23 | 1.03 | 0.87, 1.23 |
| Hyperactivity | 13.7% | 8.9% | 0.63 | 0.23, 1.77 | 0.66 | 0.24, 1.85 | 13.7% | 0.93 | 0.68, 1.27 | 0.94 | 0.69, 1.28 | 15.8% | 1.09 | 0.92, 1.28 | 1.08 | 0.92, 1.27 |
| Peer relations | 11.9% | 18.9% | 1.39 | 0.57, 3.42 | 1.45 | 0.59, 3.56 | 14.2% | 1.11 | 0.80, 1.54 | 1.11 | 0.80, 1.56 | 11.1% | 0.90 | 0.74, 1.10 | 0.90 | 0.74, 1.10 |
| Global score | 11.0% | 8.7% | 0.65 | 0.17, 2.49 | 0.67 | 0.18, 2.58 | 13.1% | 1.11 | 0.79, 1.55 | 1.10 | 0.78, 1.55 | 13.6% | 1.16 | 0.97, 1.40 | 1.17 | 0.97, 1.40 |
| **Physical complaints** |  |  |  |  |  |  |  |  |  |  |  |  |  |  |  |  |
| Frequent headaches | 50.5% | 58.1% | 1.13 | 0.76, 1.68 | 1.13 | 0.75, 1.69 | 46.1% | 0.92 | 0.80, 1.07 | 0.93 | 0.81, 1.08 | 48.1% | 0.95 | 0.89, 1.02 | 0.95 | 0.89, 1.02 |
| Abdominal pain | 11.5% | 14.7% | 1.31 | 0.48, 3.58 | 1.07 | 0.35, 3.28 | 8.9% | 0.76 | 0.51, 1.13 | 0.76 | 0.51, 1.14 | 11.8% | 1.00 | 0.82, 1.22 | 1.00 | 0.82, 1.22 |
| Constipation ^b^ | 20.8% | 14.2% | 0.71 | 0.28, 1.80 | 0.74 | 0.30, 1.84 | 22.7% | 1.08 | 0.84, 1.38 | 1.09 | 0.85, 1.39 | 22.3% | 1.07 | 0.94, 1.21 | 1.06 | 0.93, 1.21 |
| **Sleep** |  |  |  |  |  |  |  |  |  |  |  |  |  |  |  |  |
| Duration (< 9h / >12h) | 2.7% | 0.2% | 0.00 | 0.00, 0.01 | 0.00 | 0.00, 0.01 | 1.6% | 0.61 | 0.25, 1.53 | 0.62 | 0.25, 1.54 | 3.0% | 1.09 | 0.72, 1.65 | 1.10 | 0.73, 1.66 |
| Lack (Often/Always) | 13.9% | 10.9% | 0.70 | 0.19, 2.65 | 0.74 | 0.20, 2.74 | 13.6% | 0.97 | 0.70, 1.35 | 0.98 | 0.70, 1.36 | 15.4% | 1.09 | 0.93, 1.29 | 1.10 | 0.93, 1.29 |
| Difficulty falling asleep, (Always/Sometimes) | 22.8% | 12.1% | 0.41 | 0.14, 1.17 | 0.42 | 0.15, 1.20 | 20.5% | 1.03 | 0.84, 1.26 | 1.02 | 0.82, 1.25 | 23.5% | 1.03 | 0.92, 1.14 | 1.02 | 0.92, 1.13 |

The quasi Poisson regression was performed for binomial outcomes. All the binomial outcomes had categories yes/no and reference category was set as “no”, unless stated otherwise.

Analyses adjusted for: mother’s age, country of birth, education level, employment status; household income, CSP; mother’s history of diabetes mellitus, arterial hypertension, infertility treatment, pre-pregnancy BMI, smoking during pregnancy; foetal growth restriction, child’s sex;

^a^ Precocious puberty is defined as an appearance of pubic hair before the age of 9.5 for boys and 8 years for girls, or breasts before the age of 8, or period before the age of 11. ^b^ Defined as ≤ 2 stools per week;

eTable 5 Descriptive analysis (prevalence/mean (SD)) and outcome wide regression results of measured outcomes from the weighted home visit additional adjustment subsample, N = 6039

|  | **39–40 GA** | **32–33 GA, N = 26** | | | | | **34–36 GA, N = 322** | | | | | **37-38 GA, N = 1441** | | | | |
| --- | --- | --- | --- | --- | --- | --- | --- | --- | --- | --- | --- | --- | --- | --- | --- | --- |
|  | N = 4250 | *Main analysis adjusted model* | | | *+ mother’s mental health* | | *Main analysis adjusted model* | | | *+ mother’s mental health* | | *Main analysis adjusted model* | | | *+ mother’s mental health* | |
| OUTCOMES | **Mean (SD)** | **Mean (SD)** | **β** | **95% CI** | **β** | **95% CI** | **Mean (SD)** | **β** | **95% CI** | **β** | **95% CI** | **Mean (SD)** | **β** | **95% CI** | **β** | **95% CI** |
| **Anthropometry** |  |  |  |  |  |  |  |  |  |  |  |  |  |  |  |  |
| Waist/Height | 0.4 (0.1) | 0.4 (0.0) | -0.03 | -0.05, 0.00 | -0.03 | -0.06, 0.00 | 0.5 (0.0) | 0.00 | -0.01, 0.01 | 0.00 | -0.01, 0.01 | 0.5 (0.1) | 0.00 | 0.00, 0.01 | 0.00 | 0.00, 0.01 |
| WH ratio z score | 0.1 (1.1) | -0.4 (0.7) | -0.53 | -1.04, -0.01 | -0.54 | -1.16, 0.09 | 0.2 (1.0) | 0.02 | -0.14, 0.18 | 0.00 | -0.17, 0.17 | 0.2 (1.1) | 0.08 | -0.01, 0.16 | 0.10 | 0.01, 0.18 |
| Weight/age z-score: | 0.3(1.1) | 0.0 (1.0) | -0.31 | -0.77, 0.14 | -0.26 | -0.79, 0.27 | 0.3 (1.1) | -0.05 | -0.22, 0.12 | -0.01 | -0.19, 0.18 | 0.4 (1.1) | 0.09 | 0.01, 0.17 | 0.11 | 0.02, 0.19 |
| Height/age z-score | 0.3 (1.1) | 0.3 (0.8) | -0.07 | -0.43, 0.28 | -0.11 | -0.48, 0.27 | 0.3 (1.0) | -0.04 | -0.20, 0.12 | -0.03 | -0.21, 0.16 | 0.4 (1.1) | 0.06 | -0.02, 0.15 | 0.06 | -0.02, 0.15 |
| BMI/age z-score: | 0.3 (1.2) | -0.3 (1.3) | -0.47 | -1.12, 0.17 | -0.34 | -1.09, 0.40 | 0.2 (1.2) | -0.04 | -0.23, 0.15 | 0.01 | -0.19, 0.21 | 0.4 (1.2) | 0.10 | 0.01, 0.19 | 0.12 | 0.03, 0.22 |
| **BP percentiles** (mmHg) |  |  |  |  |  |  |  |  |  |  |  |  |  |  |  |  |
| Systolic | 31.6 (27.6) | 23.5 (18.8) | -8.71 | -19.15, 1.72 | -8.22 | -19.35, 2.91 | 31.9 (27.6) | -0.70 | -4.73, 3.34 | 1.08 | -3.60, 5.75 | 32.7 (29.0) | 0.37 | -1.87, 2.61 | 0.68 | -1.63, 2.99 |
| Diastolic | 51.3 (23.4) | 48.2 (21.3) | -4.00 | -13.17, 5.16 | -1.54 | -11.77, 8.70 | 51.0 (21.6) | -1.38 | -4.80, 2.04 | 1.83 | -1.77, 5.44 | 52.4 (23.2) | 0.73 | -1.09, 2.56 | 1.22 | -0.64, 3.09 |
| HR (beats/min) | 74.7 (10.6) | 74.9 (10.3) | -0.31 | -4.97, 4.35 | 2.22 | -2.74, 7.18 | 75.7 (10.9) | 0.68 | -1.05, 2.40 | 0.35 | -1.70, 2.39 | 75.7 (10.8) | 0.96 | 0.15, 1.78 | 0.93 | 0.08, 1.77 |
| **Physical fitness** |  |  |  |  |  |  |  |  |  |  |  |  |  |  |  |  |
| Jump length (m) | 1.2 (0.2) | 1.3 (0.3) | 0.06 | -0.07, 0.20 | 0.07 | -0.09, 0.23 | 1.2 (0.2) | -0.04 | -0.08, -0.01 | -0.03 | -0.07, 0.01 | 1.2 (0.2) | 0.01 | -0.01, 0.03 | 0.01 | -0.01, 0.03 |
| N of sit-ups | 11.6 (4.3) | 14.0 (4.1) | 2.33 | 0.10, 4.56 | 3.06 | 0.52, 5.60 | 11.1 (4.7) | -0.44 | -1.12, 0.24 | -0.56 | -1.31, 0.19 | 11.5 (4.6) | 0.02 | -0.33, 0.37 | 0.00 | -0.35, 0.36 |
| **Cognitive functioning** |  |  |  |  |  |  |  |  |  |  |  |  |  |  |  |  |
| Matrix FSIQ score ^a^ | 10.0 (2.5) | 9.8 (2.4) | -0.15 | -1.57, 1.27 | -0.08 | -1.83, 1.67 | 9.9 (2.3) | 0.09 | -0.28, 0.45 | 0.07 | -0.35, 0.49 | 9.8 (2.6) | -0.04 | -0.24, 0.16 | 0.02 | -0.18, 0.22 |
| Puzzle PIQ score ^a^ | 10.5 (2.5) | 9.5 (2.7) | -0.87 | -2.10, 0.37 | -1.14 | -2.58, 0.29 | 10.2 (2.3) | -0.02 | -0.37, 0.33 | -0.03 | -0.44, 0.37 | 10.3 (2.4) | -0.13 | -0.32, 0.05 | -0.08 | -0.27, 0.11 |
| PPVT score ^b^ | 98.0 (12.8) | 101.3 (11.6) | 3.38 | -2.04, 8.80 | 1.83 | -3.93, 7.58 | 96.4 (14.4) | -0.35 | -2.54, 1.85 | -0.30 | -2.92, 2.32 | 97.1 (12.7) | -0.17 | -1.10, 0.76 | 0.02 | -0.94, 0.99 |
|  |  |  |  |  |  |  |  |  |  |  |  |  |  |  |  |  |
|  | **%** | **%** | **RR** | **95% CI** | **RR** | **95% CI** | **%** | **RR** | **95% CI** | **RR** | **95% CI** | **%** | **RR** | **95% CI** | **RR** | **95% CI** |
| **Motor skills** |  |  |  |  |  |  |  |  |  |  |  |  |  |  |  |  |
| Dribbling (0-1/4) | 19.0% | 8.1% | 0.72 | 0.42, 1.23 | 0.36 | 0.08, 1.57 | 22.1% | 0.93 | 0.66, 1.32 | 0.92 | 0.63, 1.34 | 21.7% | 1.12 | 0.96, 1.31 | 1.12 | 0.95, 1.31 |
| One leg (0-2/5) | 5.5% | 15.5% | 2.05 | 0.92, 4.54 | 3.59 | 0.98, 13.08 | 10.8% | 1.73 | 1.04, 2.87 | 1.68 | 0.97, 2.89 | 6.3% | 1.14 | 0.83, 1.56 | 1.13 | 0.83, 1.56 |
| Throwing a ball (0-1/4) | 23.8% | 32.5% | 1.04 | 0.68, 1.60 | 1.30 | 0.60, 2.84 | 28.0% | 1.03 | 0.77, 1.37 | 1.00 | 0.73, 1.37 | 25.1% | 1.03 | 0.90, 1.19 | 1.03 | 0.90, 1.18 |
| Jumping (0-1/4) | 10.8% | 5.7% | 0.89 | 0.45, 1.77 | 0.32 | 0.03, 3.23 | 13.1% | 1.05 | 0.65, 1.69 | 1.02 | 0.60, 1.72 | 14.6% | 1.34 | 1.08, 1.67 | 1.34 | 1.08, 1.67 |
| Global score (0-8/17) | 3.5% | 3.1% | 1.38 | 0.56, 3.40 | 0.99 | 0.10, 9.28 | 5.9% | 1.39 | 0.68, 2.88 | 1.43 | 0.66, 3.09 | 4.9% | 1.40 | 0.94, 2.09 | 1.40 | 0.94, 2.09 |

Linear regression was performed for continuous outcomes (β, 95% CI), quasi Poisson regression for binomial outcomes (RR, 95% CI). Analyses adjusted for: mother’s age, country of birth, education level, employment status; household income, CSP; mother’s history of diabetes mellitus, arterial hypertension, infertility treatment, pre-pregnancy BMI, smoking during pregnancy; foetal growth restriction, child’s sex;

^a^ Standard score (mean = 10, SD = 3) from the matrix subtest of Fluid Reasoning Index (FRI) and puzzle subtest of the Visual Spatial Index (VSI) of the WISC-V; ^b^ Peabody Picture Vocabulary Test (PPVT) 5, adapted to French, assessing children’s knowledge of spoken words and receptive vocabulary;
