# Supplementary material for: Health and Development of Children Born Moderate and Late Preterm and Early Term at Age 10 in French Birth Cohorts ELFE and EPIPAGE 2
Source: Paediatr Perinat Epidemiol. 2025 Sep 29;40(1):34–52. doi: 10.1111/ppe.70069 (PMC12853227; doi:10.1111/ppe.70069)
Supplement: Supplementary file 1 — Data S1: ppe70069‐sup‐0001‐Supinfo01.zip. [file PPE-40-34-s001.zip › Appendix 5 Table of attrition by gestational age.docx]

**Appendix 5**

| Table of attrition by gestational age with percentages of the ELFE and EPIPAGE-2 participants lost by the 10-year follow-up | | | | | | | | | | |
| --- | --- | --- | --- | --- | --- | --- | --- | --- | --- | --- |
|  | 32 GA | 33 GA | 34 GA | 35 GA | 36 GA | 37 GA | 38 GA | 39 GA | 40 GA | NA |
| **Inclusion (N)** | 247 | 409 | 699 | 242 | 497 | 1137 | 2591 | 5012 | 4961 | 392 |
| **Telephone interview (N)** | 120 | 205 | 330 | 111 | 229 | 554 | 1311 | 2651 | 2418 | 144 |
| % lost | *51.5%* | *49.9%* | *52.8%* | *54.1%* | *54.0%* | *51.3%* | *49.4%* | *47.3%* | *51.3%* | *63.3%* |
| **Home visit (N)** | 86 | 142 | 216 | 92 | 181 | 400 | 1015 | 2045 | 2125 | 116 |
| % lost | *65.2%* | *65.3%* | *69.1%* | *62.0%* | *63.6%* | *64.8%* | *60.8%* | *59.2%* | *57.2%* | *69.4%* |
